# Supplementary material for: In vitro activity of aryl-thiazole derivatives against Schistosoma mansoni schistosomula and adult worms
Source: PLoS One. 2019 Nov 25;14(11):e0225425. doi: 10.1371/journal.pone.0225425 (PMC6876889; doi:10.1371/journal.pone.0225425)
Supplement: S1 Text — Supplementary Figs A to H. Fig A, ATP quantitation using a luminescent assay to assess schistosomula survival under NJ series compounds exposure; Fig B, Effect of NJ series compounds on the viability of S. mansoni adult worms; Fig C, Effect of NJ series compounds on the motility of S. mansoni adult worms; Fig D, In vitro effect of NJ series compounds on pairing of S. mansoni adult worm couples; Fig E, In vitro effects of NJ series compounds on oviposition of S. mansoni adult worms; Fig F, Cytotoxicity evaluation of exposure of two different human cell lines to NJ series compounds; Fig G, Structural damage was observed by scanning electron microscopy of male worms already at 24 h exposure to NJ05 or NJ07; Fig H, Vitellaria and female reproduction gene expression in adult females upon NJ07 or NJ05+NJ07 treatment. (PDF) [file pone.0225425.s001.pdf]

S1 Text. Supplementary Figs A to H.

Supporting Information for

In vitro activity of aryl-thiazole derivatives against *Schistosoma mansoni* schistosomula and adult worms

Adriana SA Pereira[1,2], Gilbert O Silveira[1,2], Murilo S Amaral[1], Sinara MV Almeida[3,4], Jamerson F Oliveira[3], Maria CA Lima[3], Sergio Verjovski-Almeida[1,2]

[1] Instituto Butantan, São Paulo, Brasil.

[2] Departamento de Bioquímica, Instituto de Química, Universidade de São Paulo, São Paulo, Brasil.

[3] Universidade Federal de Pernambuco, Departamento de Antibióticos, Recife, Pernambuco, Brasil.

[4] Universidade de Pernambuco, campus Garanhuns, Garanhuns, Pernambuco, Brasil.

Corresponding author: Sergio Verjovski-Almeida - verjo@iq.usp.br

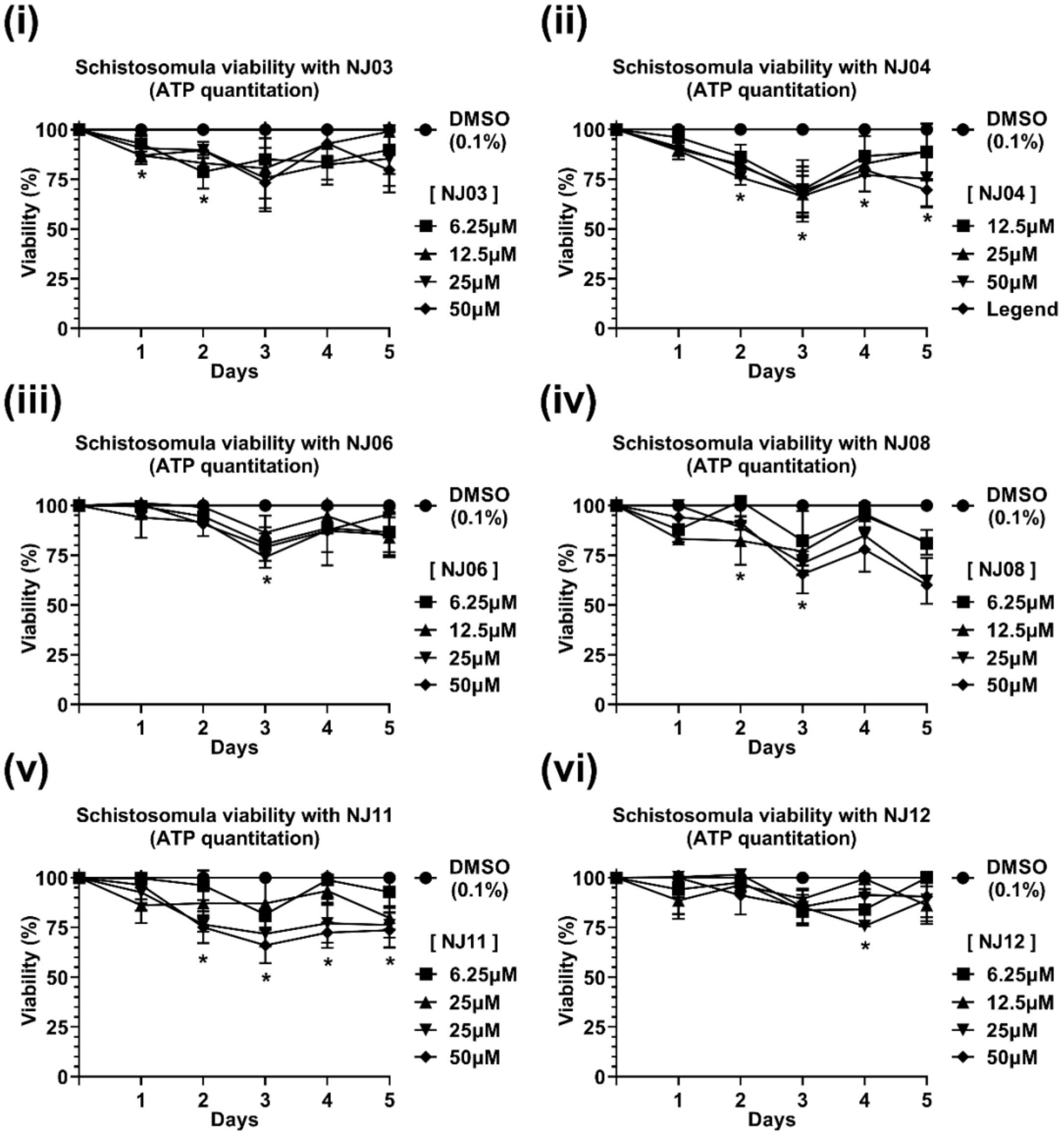

Fig A. ATP quantitation using a luminescent assay to assess schistosomula survival under NJ series compounds exposure. Schistosomula (100-120/well) were incubated with the indicated concentrations of NJ03, NJ04, NJ06, NJ08, NJ11 or NJ12 or with control vehicle (0.1% DMSO) for up to 5 days. Viability is expressed as % luminescence values relative to the control (DMSO). Mean ± SEM from three replicate experiments. *$p < 0.05$ (two-way ANOVA). For clarity purposes, we show only the highest p-value obtained from the two-way ANOVA test for each time point on all different concentrations tested.

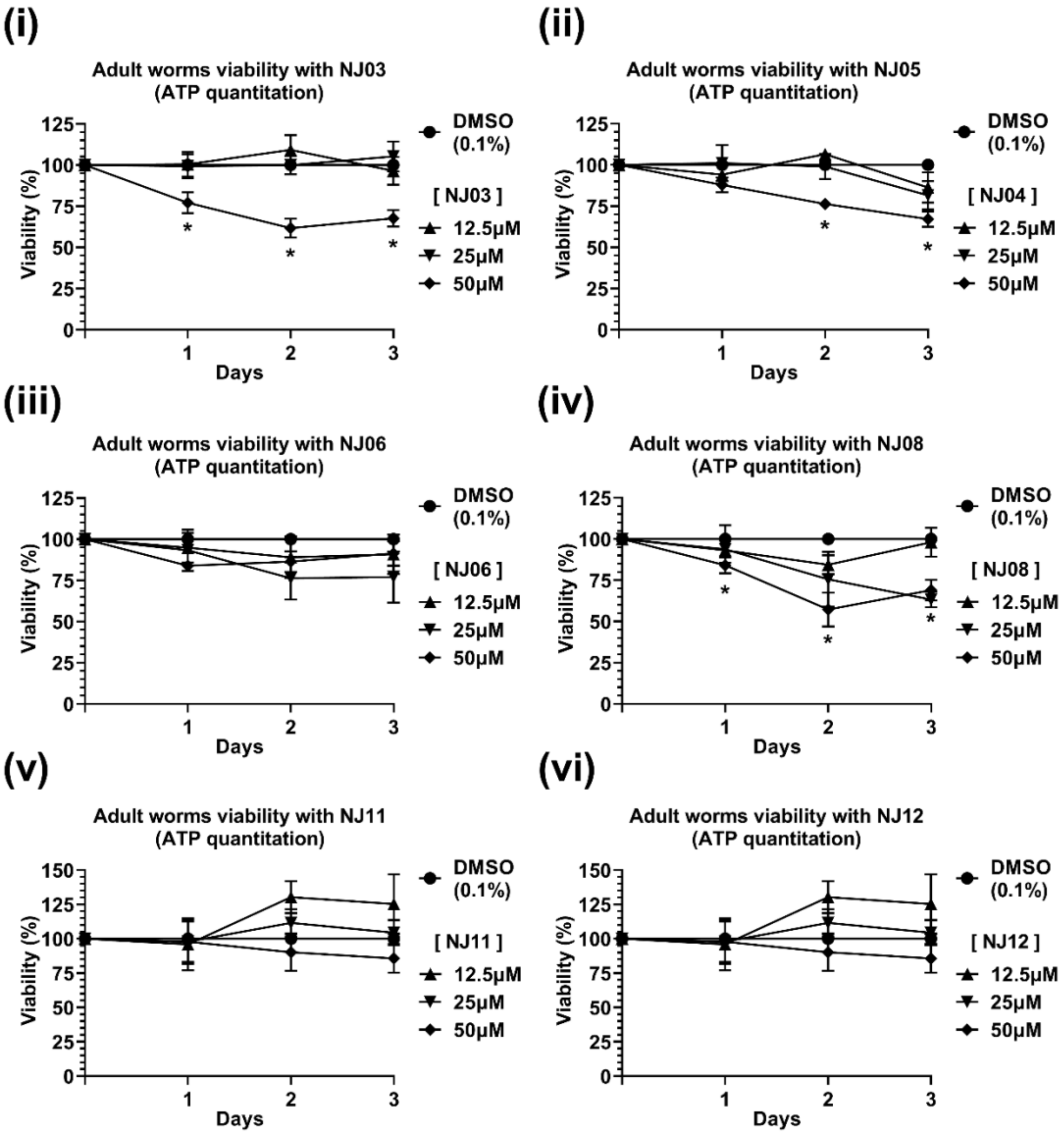

Fig B. Effect of NJ series compounds on the viability of *Schistosoma mansoni* adult worms. Viability was estimated by the total amount of ATP available in the parasites, using a luminescent assay. Pairs of adult worms were treated for 1 to 3 days with NJ03, NJ04, NJ06, NJ08, NJ12 and NJ12 at the different concentrations indicated or with vehicle (0.1% DMSO). Viability was expressed as % luminescence values relative to the control (0.1% DMSO). Mean ± SEM from three replicate experiments, each with 10 worm pairs. *p < 0.05 compared with control. For clarity purposes, we show only the highest p-value obtained from the two-way ANOVA test for each time point on all different concentrations tested.

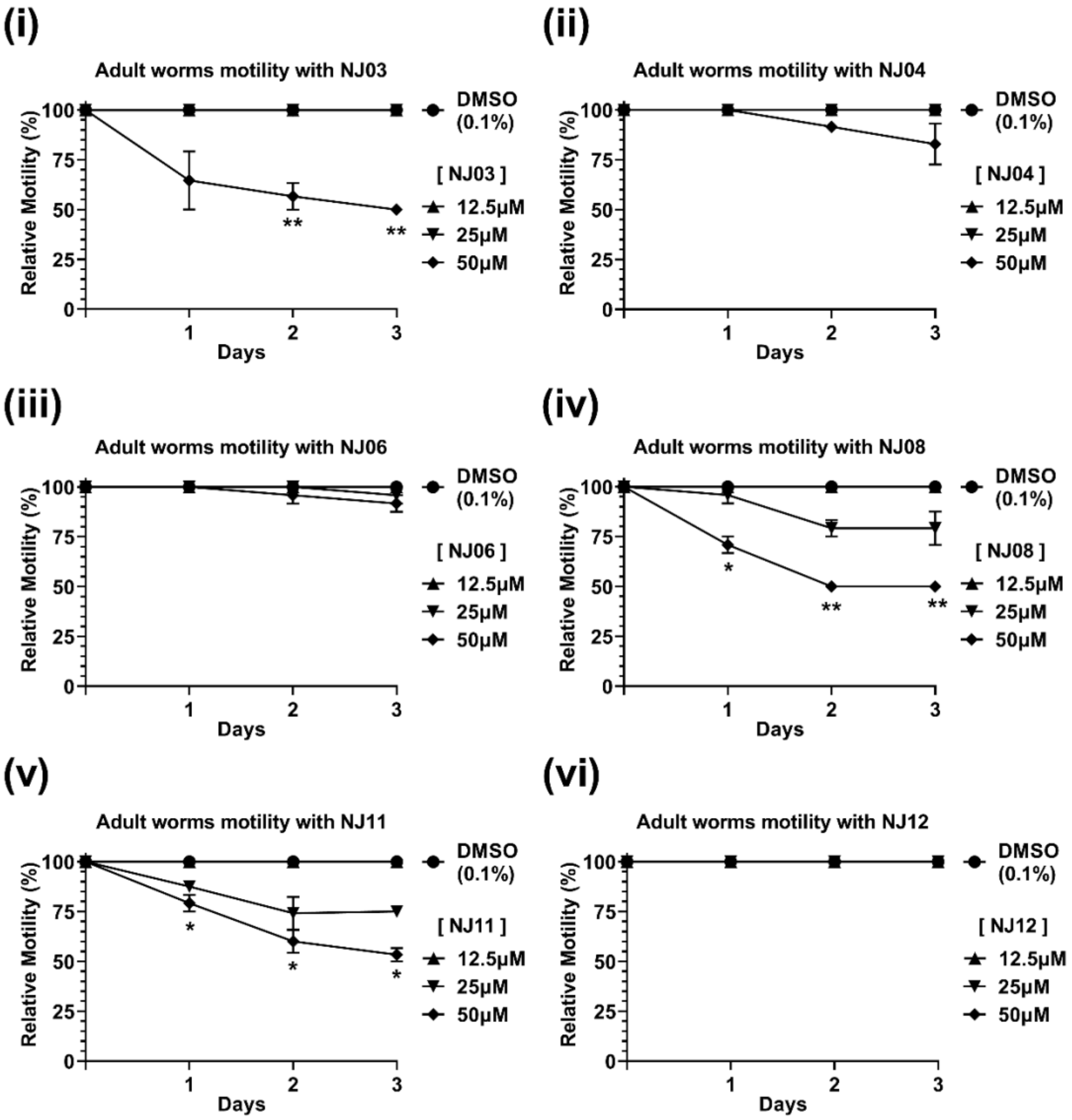

Fig C. Effect of NJ series compounds on the motility of *Schistosoma mansoni* adult worms. Percentage of relative motility of adult worms treated with different concentrations of NJ03, NJ04, NJ06, NJ08, NJ12 and NJ12 and controls (0.1 % DMSO) at different times of exposure from 1 to 3 days. Mean ± SEM of three experiments, each with 10 worm pairs. *p < 0.05, **p < 0.01 compared with control. For clarity purposes, we show only the highest p-value obtained from the two-way ANOVA test for each time point on all different concentrations tested.

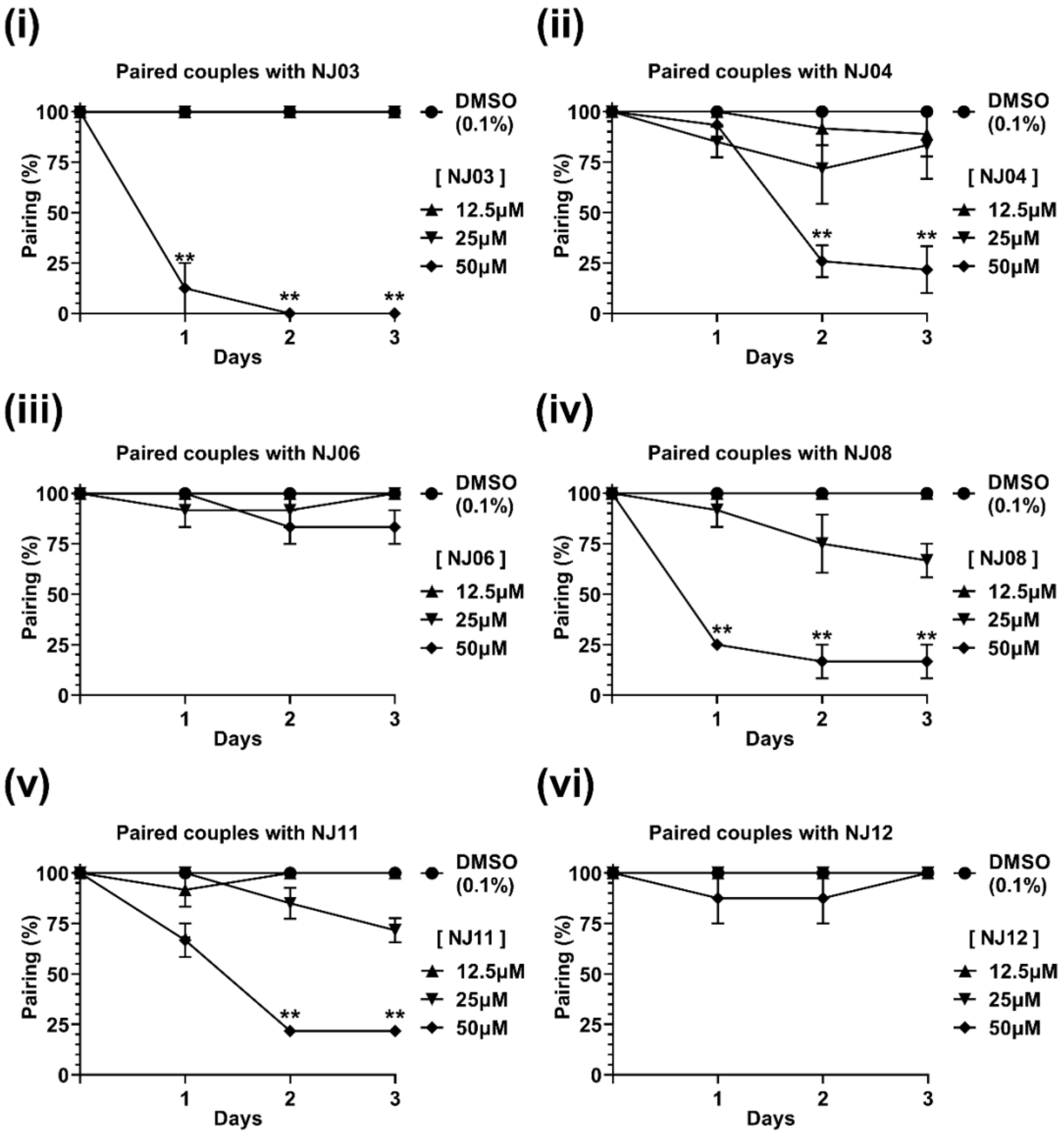

Fig D. *In vitro* effect of NJ series compounds on pairing of *Schistosoma mansoni* adult worm couples. We monitored the pairing status of control couples and of treated couples exposed to different concentrations of NJ03, NJ04, NJ06, NJ08, NJ11 and NJ12 at different times of exposure from 1 to 3 days. Mean ± SEM of three experiments, each with 10 worm pairs. *p < 0.05 and **p < 0.01 compared with control. For clarity purposes, we show only the highest p-value obtained from the two-way ANOVA test for each time point on all different concentrations tested.

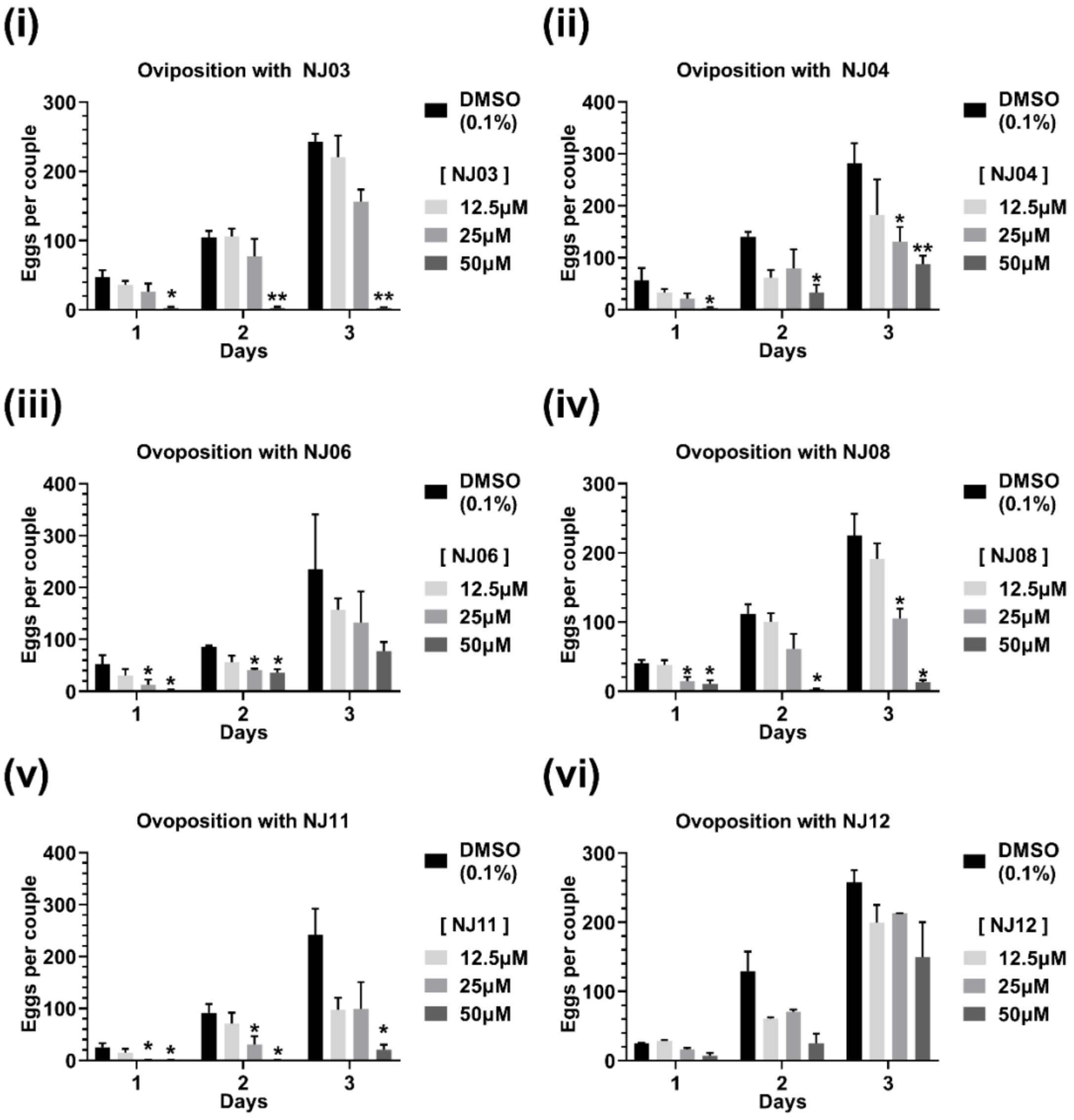

Fig E. *In vitro* effects of NJ series compounds on oviposition of *Schistosoma mansoni* adult worms. Number of eggs released by females incubated with NJ03, NJ04, NJ06, NJ08, NJ11 and NJ12 at different concentrations or with vehicle (0.1 % DMSO) at different times of exposure from 1 to 3 days. Mean ± SEM of three experiments, each with 10 worm pairs. *p < 0.05 and **p < 0.01 compared with control. For clarity purposes, we show only the highest p-value obtained from the two-way ANOVA test for each time point on all different concentrations tested.

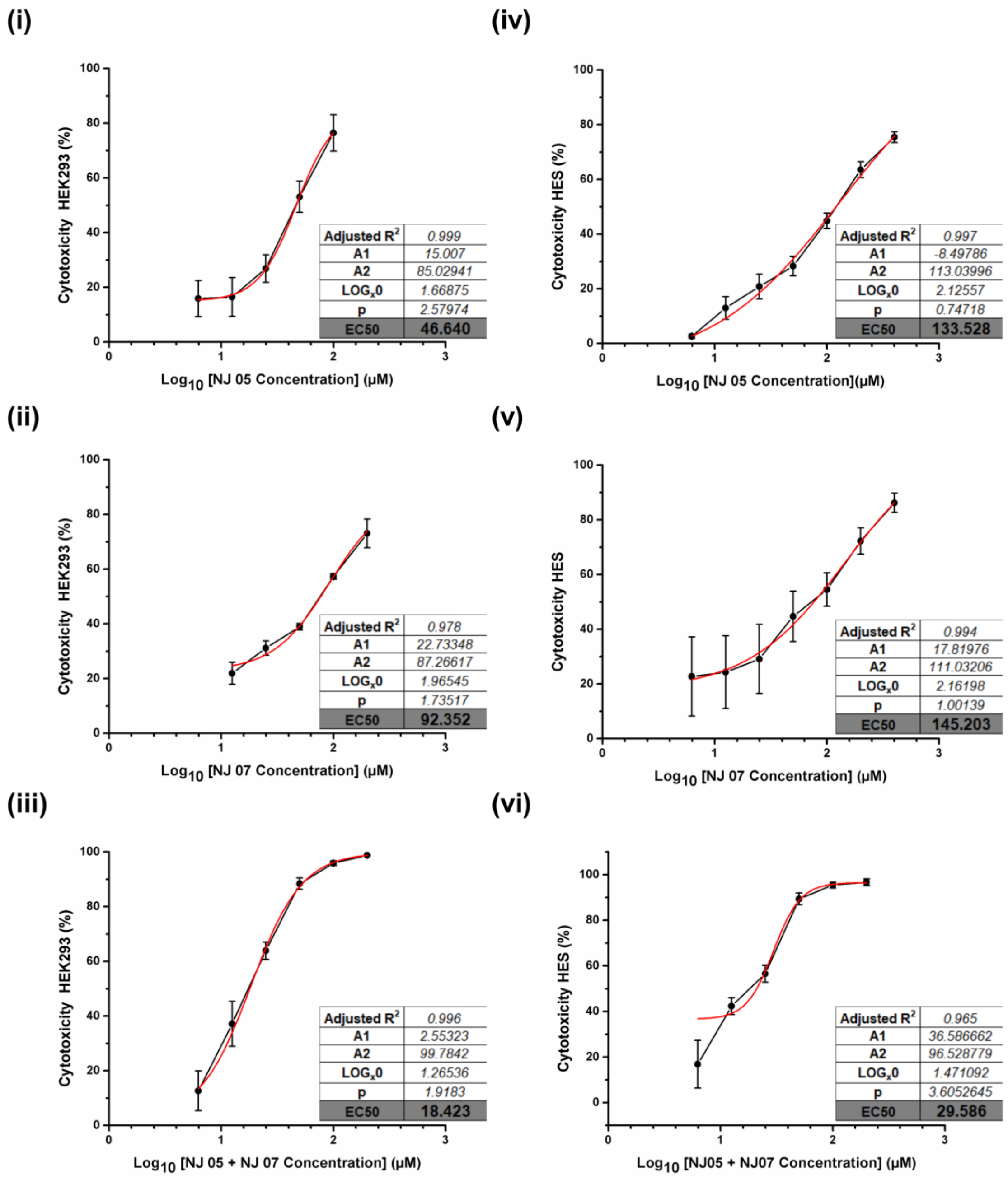

Fig F. Cytotoxicity evaluation of exposure of two different human cell lines to NJ series compounds. HEK293 (Human Embryo Kidney- HEK-293 -ATCC CRL-1573) (left panels) and HES (human endometrial epithelial) cell lines (right panels) were exposed to different concentrations of NJ05, NJ07, NJ05+NJ07 or to vehicle (0.1 % DMSO). After two days of exposure the cells (5x10^{3} cells/well) had their viability measured by ATP quantitation and cytotoxicity was evaluated. The equation used to fit

the cytotoxicity values was y = A1+(A2-A1)/(1+10)^((LOGx0-x)*p)). The fitted equation parameters as well as the adjusted R-square are represented at the insets, with the EC50 for each compound highlighted in grey.

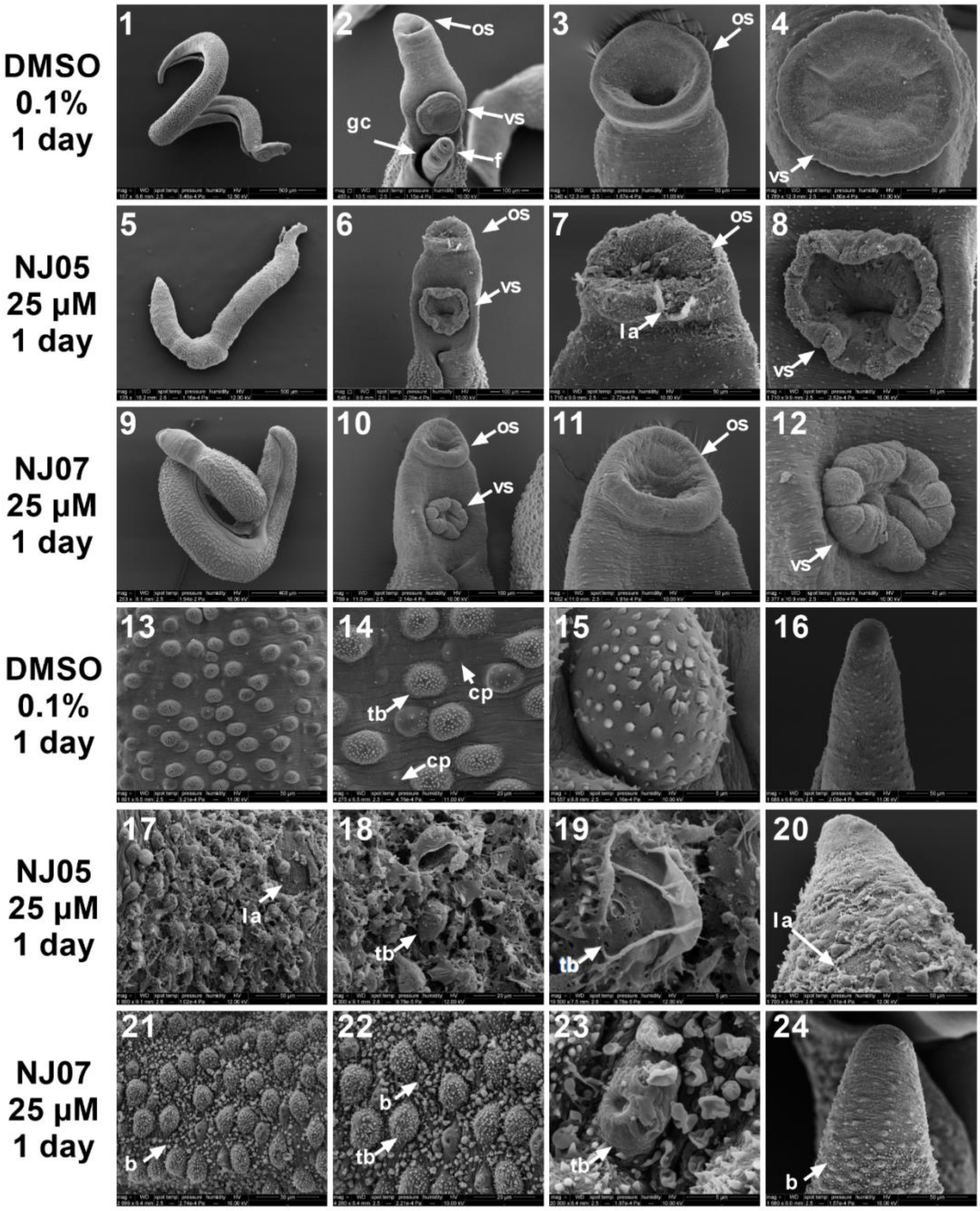

Fig G. Structural damage was observed by scanning electron microscopy of male worms already at 24 h exposure to NJ05 or NJ07. Worms were exposed for 24 h to 25 µM NJ05, 25 µM NJ07 or to vehicle (0.1% DMSO). **panels 1-4**: Control worms (Bar = 500 µm; 100 µm; 50 µm; 50 µm) presented the fully paired couple (panel 2) and anterior region of the male presented normal oral and ventral suckers tegument (panels

3 and 4); **panels 5-8**: Anterior region of adult male worm treated with NJ05 with oral and ventral suckers presented tegument peeling (Bar = 500 μm; 100 μm; 50 μm; 50 μm); **panels 9-12**: Anterior region of adult male worm treated with NJ07 with oral and ventral suckers presented tegument without structural alterations (Bar = 400 μm; 100 μm; 50 μm; 40 μm); **panels 13-16**: Medial (panels 13 – 15) and posterior (panel 16) regions of control male worm presented normal tegument structures (Bar = 50 μm; 20 μm; 5 μm; 50 μm); **panels 17-20:** Enlarged view of dorsal region of adult worm treated with NJ05 (panels 17-19) showed tegument lesions and loss of tubercles and posterior region of male adult worm showed lesion areas (Bar = 50 μm; 20 μm; 5 μm; 50 μm); **panels 21-24:** Dorsal and posterior regions of adult worm treated with NJ07 showed bubbles along their extension (Bar = 30 μm; 20 μm; 5 μm; 50 μm). *f*: *female; os*: *oral sucker; vs*: *ventral sucker; gc: gynecophoral canal*; *la*: *lesion area* ; *cp: ciliated papillae; b: blebs; tb*: *tubercles*. (note that the size scale bar is shown within the black thin line below each image).

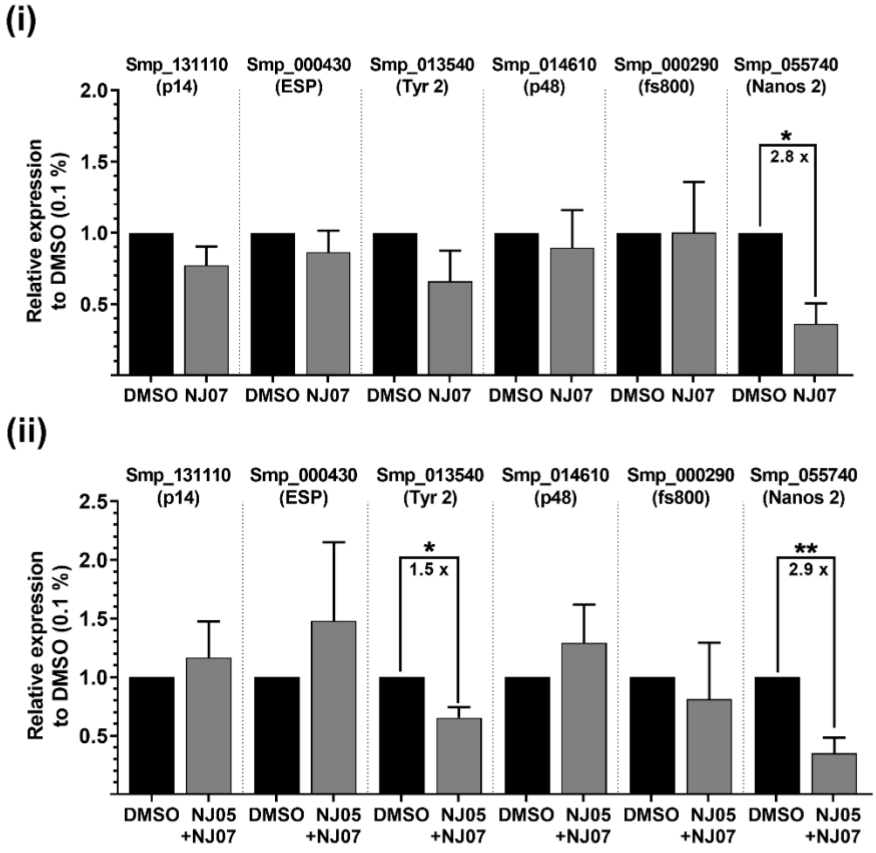

Fig H: Vitellaria and female reproduction gene expression in adult females upon NJ07 or NJ05+NJ07 treatment. Adult couples were treated with NJ07 (25 µM), NJ05+NJ07 (25 µM) or DMSO (0.1 %) for 2 days. The parasites were stored in RNAlater for further processing. All parasite couples were separated and only the females had their RNA extracted followed by cDNA synthesis. The genes measured by qPCR were Smp_131110 (p14), Smp_000430 (Egg Shell Protein (ESP)), Smp_013540 (Tyrosinase 2 (Tyr 2)), Smp_014610 (p48), Smp_000290 (fs800), Smp_055740 (Nanos 2). The geometric mean from two reference genes was used for normalization with the DCT method (Smp_090920 and Smp_123610). The plotted data is retrieved from DDCT analyses in which DMSO sample was the control. Significant fold-change in gene expression between DMSO and NJ07 or NJ05+NJ07 treatment is shown by the numbers inside the brackets. Student unpaired parametric two-sided t-test was used

and statistically significant differences are represented by the asterisks. *$p \leq 0.05$; **$p \leq 0.01$.
