# Supplementary material for: In vitro activity of aryl-thiazole derivatives against Schistosoma mansoni schistosomula and adult worms
Source: PLoS One. 2019 Nov 25;14(11):e0225425. doi: 10.1371/journal.pone.0225425 (PMC6876889; doi:10.1371/journal.pone.0225425)
Supplement: Attachment — Submitted filename: PONE-D-19-21297_revised manuscript.pdf [file pone.0225425.s003.pdf]

PLOS ONE

In vitro activity of aryl-thiazole derivatives against Schistosoma mansoni schistosomula and adult worms

--Manuscript Draft--

**Manuscript Number:**	PONE-D-19-21297
**Article Type:**	Research Article
**Full Title:**	In vitro activity of aryl-thiazole derivatives against Schistosoma mansoni schistosomula and adult worms
**Short Title:**	Activity evaluation of thiazole derivatives on Schistosoma mansoni
**Corresponding Author:**	Sergio Verjovski-Almeida<br>Instituto de Quimica Universidade de Sao Paulo<br>Sao Paulo, SP BRAZIL
**Keywords:**	Schistosoma, Schistosoma mansoni, aryl-thiazole, thiazole, in vitro drug treatment, parasites viability, parasites egg deposition
**Abstract:**	Background:<br>Schistosomiasis is a chronic and debilitating disease caused by a trematode of the genus Schistosoma affecting over 200 million people worldwide. Current strategy for treatment and control of schistosomiasis is the use of Praziquantel, the only drug recommended by the World Health Organization. Development of new drugs is therefore of great importance. Thiazoles are regarded as privileged structures with a broad spectrum of activities and are potential sources of new drug prototypes, since they can act through interactions with DNA and inhibition of DNA synthesis.<br>Methodology/Principal findings:<br>In this context, we report the synthesis of a series of thiazole derivatives and the in vitro schistosomicidal testing 8 molecules (NJ03-08; NJ11-12) containing these structures. Parameters such as motility and mortality, ~~egg laying~~, ~~pairing~~ and parasite viability by ATP quantification, which were influenced by these compounds, were evaluated during the assays. Scanning electron microscopy (SEM) was utilized for evaluation of morphological changes in the tegument. Adult worms and schistosomula were treated in vitro with different concentrations (6.25 to 50 µM) of the thiazoles for up to 3 and 5 days, respectively. Adult worms after three days with 50 µM NJ05, NJ07 or NJ05 + NJ07 showed decreased motility to 30–50 % compared to controls. Compound NJ05 was more effective than NJ07, and viability after three days was reduced to 25 % in parasites treated with 50 µM NJ05, compared with a viability reduction to 40 % with 50 µM NJ07. SEM analysis showed ~~severe~~ alterations in adult worms with formation of ~~bulges and~~ blisters throughout the dorsal region of parasites treated with NJ05 and NJ07. Oviposition was ~~extremely~~ affected by treatment with the NJ series compounds; at concentrations of 25 µM and 50 µM, oviposition reached almost zero with NJ05, NJ07 and NJ05 + NJ07 already at day one. Tested genes involved in egg biosynthesis were all confirmed by qPCR as downregulated in females treated with 25 µM NJ05 for 2 days, with a significant reduction in expression of p14, Tyrosinase 2, p48 and fs800.<br>Conclusions/Significance:<br>According to the present study, thiazole derivatives have schistosomicidal activities and may be part of a possible new arsenal of compounds against schistosomiasis.
**Order of Authors:**	Adriana S A Pereira
	Gilbert O Silveira
	Murilo S Amaral
	Sinara M V Almeida
	Jamerson F Oliveira
	Maria C A Lima
	Sergio Verjovski-Almeida
**Additional Information:**	

Powered by Editorial Manager® and ProduXion Manager® from Aries Systems Corporation

Question	Response
**Financial Disclosure**<br><br>Enter a financial disclosure statement that describes the sources of funding for the work included in this submission. Review the submission guidelines for detailed requirements. View published research articles from *PLOS ONE* for specific examples.<br><br>This statement is required for submission and **will appear in the published article** if the submission is accepted. Please make sure it is accurate.<br><br>**Unfunded studies**<br>Enter: *The author(s) received no specific funding for this work.*<br><br>**Funded studies**<br>Enter a statement with the following details:<br>• Initials of the authors who received each award<br>• Grant numbers awarded to each author<br>• The full name of each funder<br>• URL of each funder website<br>• Did the sponsors or funders play any role in the study design, data collection and analysis, decision to publish, or preparation of the manuscript?<br>• **NO** - Include this sentence at the end of your statement: *The funders had no role in study design, data collection and analysis, decision to publish, or preparation of the manuscript.*<br>• **YES** - Specify the role(s) played.<br><br>* typeset	This work was supported in by a grant from Fundação de Amparo à Pesquisa do Estado de São Paulo (FAPESP) to SVA. Fellowships from FAPESP have supported ASAP (2016/10046-6) and GOS (2018/24015-0). MSA was supported by Fundação Butantan. SMVA, JFO and MCAL were supported by fellowship PNPD from Coordenação de Aperfeiçoamento de Pessoal de Nível Superior (CAPES), process BCT-0149-4.03/17. SVA was also supported by institutional funds from Fundação Butantan. SVA is the recipient of an established investigator fellowship award from Conselho Nacional de Desenvolvimento Cientifico e Tecnologico, Brasil. The funders had no role in study design, data collection and analysis, decision to publish or preparation of the manuscript.
**Competing Interests**<br><br>Use the instructions below to enter a competing interest statement for this submission. On behalf of all authors, disclose any competing interests that could be perceived to bias this work—acknowledging all financial support and any other relevant financial or non-financial competing interests.<br><br>This statement **will appear in the published article** if the submission is	The authors have declared that no competing interests exist

accepted. Please make sure it is accurate. View published research articles from *PLOS ONE* for specific examples.<br><br>**NO authors have competing interests**<br><br>Enter: *The authors have declared that no competing interests exist.*<br><br>**Authors with competing interests**<br><br>Enter competing interest details beginning with this statement:<br><br>*I have read the journal's policy and the authors of this manuscript have the following competing interests: [insert competing interests here]*<br><br>* typeset	
**Ethics Statement**<br><br>Enter an ethics statement for this submission. This statement is required if the study involved:<br><br>• Human participants<br>• Human specimens or tissue<br>• Vertebrate animals or cephalopods<br>• Vertebrate embryos or tissues<br>• Field research<br><br>Write "N/A" if the submission does not require an ethics statement.<br><br>General guidance is provided below. Consult the submission guidelines for detailed instructions. **Make sure that all information entered here is included in the Methods section of the manuscript.**	The experimental protocols were in accordance with the Ethical Principles in Animal Research adopted by the Brazilian College of Animal Experimentation (COBEA) and the protocol/experiments have been approved by the Ethics Committee for Animal Experimentation of Institute Butantan (CEUA Nº 1777050816).

**Format for specific study types**<br><br>**Human Subject Research (involving human participants and/or tissue)**<br>• Give the name of the institutional review board or ethics committee that approved the study<br>• Include the approval number and/or a statement indicating approval of this research<br>• Indicate the form of consent obtained (written/oral) or the reason that consent was not obtained (e.g. the data were analyzed anonymously)<br><br>**Animal Research (involving vertebrate animals, embryos or tissues)**<br>• Provide the name of the Institutional Animal Care and Use Committee (IACUC) or other relevant ethics board that reviewed the study protocol, and indicate whether they approved this research or granted a formal waiver of ethical approval<br>• Include an approval number if one was obtained<br>• If the study involved *non-human primates*, add *additional details* about animal welfare and steps taken to ameliorate suffering<br>• If anesthesia, euthanasia, or any kind of animal sacrifice is part of the study, include briefly which substances and/or methods were applied<br><br>**Field Research**<br><br>Include the following details if this study involves the collection of plant, animal, or other materials from a natural setting:<br>• Field permit number<br>• Name of the institution or relevant body that granted permission	
**Data Availability**<br><br>Authors are required to make all data underlying the findings described fully available, without restriction, and from the time of publication. PLOS allows rare exceptions to address legal and ethical concerns. See the PLOS Data Policy and FAQ for detailed information.	Yes - all data are fully available without restriction

A Data Availability Statement describing where the data can be found is required at submission. Your answers to this question constitute the Data Availability Statement and **will be published in the article**, if accepted.<br><br>**Important:** Stating 'data available on request from the author' is not sufficient. If your data are only available upon request, select 'No' for the first question and explain your exceptional situation in the text box.<br><br>Do the authors confirm that all data underlying the findings described in their manuscript are fully available without restriction?	
**Describe where the data may be found in full sentences. If you are copying our sample text, replace any instances of XXX with the appropriate details.**<br><br>• If the data are **held or will be held in a public repository**, include URLs, accession numbers or DOIs. If this information will only be available after acceptance, indicate this by ticking the box below. For example: *All XXX files are available from the XXX database (accession number(s) XXX, XXX.).*<br>• If the data are all contained **within the manuscript and/or Supporting Information files**, enter the following: *All relevant data are within the manuscript and its Supporting Information files.*<br>• If neither of these applies but you are able to provide **details of access elsewhere**, with or without limitations, please do so. For example:<br><br>*Data cannot be shared publicly because of [XXX]. Data are available from the XXX Institutional Data Access / Ethics Committee (contact via XXX) for researchers who meet the criteria for access to confidential data.*<br><br>*The data underlying the results presented in the study are available from (include the name of the third party*	All relevant data are within the manuscript and its Supporting Information files.

and contact information or URL).<br>• This text is appropriate if the data are owned by a third party and authors do not have permission to share the data.<br><br>* typeset	
Additional data availability information:	

Manuscript
Click here to access/download;Manuscript;NJ paper submitted PLOS NTD (06-18-19) Pereira et al.docx

In vitro activity of aryl-thiazole derivatives against *Schistosoma mansoni* schistosomula and adult worms

Adriana SA Pereira[1,2], Gilbert O Silveira[1,2], Murilo S Amaral[1], Sinara MV Almeida[3,4], Jamerson F Oliveira[3], Maria CA Lima[3], Sergio Verjovski-Almeida[1,2]

[1] Instituto Butantan, São Paulo, Brasil.

[2] Departamento de Bioquímica, Instituto de Química, Universidade de São Paulo, São Paulo, Brasil.

[3] Universidade Federal de Pernambuco, Departamento de Antibióticos, Recife, Pernambuco, Brasil.

[4] Universidade de Pernambuco, campus Garanhuns, Garanhuns, Pernambuco, Brasil.

Short title: Activity evaluation of thiazole derivatives on *Schistosoma mansoni*

Corresponding author: Sergio Verjovski-Almeida - verjo@iq.usp.br

Abstract:

Background:

Schistosomiasis is a chronic and debilitating disease caused by a trematode of the genus *Schistosoma* affecting over 200 million people worldwide. Current strategy for treatment and control of schistosomiasis is the use of Praziquantel, the only drug recommended by the World Health Organization. Development of new drugs is therefore of great importance. Thiazoles are regarded as privileged structures with a broad spectrum of activities and are potential sources of new drug prototypes, since they can act through interactions with DNA and inhibition of DNA synthesis.

Methodology/Principal findings:

In this context, we report the synthesis of a series of thiazole derivatives and the *in vitro* schistosomicidal testing 8 molecules (NJ03-08; NJ11-12) containing these structures. Parameters such as motility and mortality, ~~egg laying, pairing~~ and parasite viability by ATP quantification, which were influenced by these compounds, were evaluated during the assays. Scanning electron microscopy (SEM) was utilized for evaluation of morphological changes in the tegument. Adult worms and schistosomula were treated *in vitro* with different concentrations (6.25 to 50 µM) of the thiazoles for up to 3 and 5 days, respectively. Adult worms after three days with 50 µM NJ05, NJ07 or NJ05 + NJ07 showed decreased motility to 30–50 % compared to controls. Compound NJ05 was more effective than NJ07, and viability after three days was reduced to 25 % in parasites treated with 50 µM NJ05, compared with a viability reduction to 40 % with 50 µM NJ07. SEM analysis showed severe alterations in adult worms with formation of bulges and blisters throughout the dorsal region of parasites treated with NJ05 and NJ07. Oviposition was ~~extremely~~ affected by treatment with the NJ series compounds; at concentrations of 25 µM and 50 µM, oviposition reached almost zero with NJ05, NJ07 and NJ05 + NJ07 already at day one. Tested genes involved in egg biosynthesis were all confirmed by qPCR as downregulated in females treated with 25 µM NJ05 for 2 days, with a significant reduction in expression of p14, Tyrosinase 2, p48 and fs800.

Conclusions/Significance:

According to the present study, thiazole derivatives have schistosomicidal activities and may be part of a possible new arsenal of compounds against schistosomiasis.

Keywords: ~~Schistosoma,~~ *Schistosoma mansoni*, aryl-thiazole, thiazole, *in vitro* drug treatment, ~~parasites viability, parasites egg deposition~~

Author Summary

Schistosomiasis is a neglected tropical disease considered a serious public health problem caused by Schistosoma worms, praziquantel (PZQ) is the only drug used to treat humans, but selection of resistant parasites after prolonged treatment in a population has been reported. Faced with this panorama and the severity of the disease, the search for new drug candidates with schistosomicidal activity is pressing. A class of aryl-thiazole derivatives has gained attention because of its promising experimental anti-schistosomal activity against schistosomula and adult worms of *Schistosoma mansoni*. In this context, our research group synthesized a series of eight new thiazole derivatives (NJ03-08; NJ11-12) as candidate schistosomicidal agents. Molecules NJ05 and NJ07 stood out, leading to a higher mortality of worms, with a significant decline in motility, ~~inhibition of pairing~~ and oviposition and causing significant ultrastructural alterations such as surface peeling and bubbles formation, with destruction of the tegument in both male and female worms. Compounds NJ05 and

NJ07 together showed promising activity against early-developing larval forms. In females treated with NJ05, a marked decrease was observed in the expression of genes related to egg formation and cell differentiation. In conclusion, the aryl-thiazoles act as a molecular framework that has antischistosomal activity, being a promising target for developing novel drugs against schistosomiasis.

INTRODUCTION

Schistosomiasis is recognized as a serious public health problem in the world [1, 2]. According to the World Health Organization (WHO), it is estimated that more than 206 million people worldwide are affected by the disease and that more than 700 million people live in areas where the disease is endemic [3]. Schistosomiasis is an infection caused by a trematode of the genus Schistosoma and occupies, after malaria, the second position in the world among parasitic infections [4, 5]. This chronic disease is prevalent in tropical and subtropical regions, mainly in regions that lack basic sanitation, allowing the proliferation of contaminating agents, as well as the continuity of people being infected [2, 3, 6]. In addition, the absence of campaigns that promote health education to the population, as well as the lack of dissemination of epidemiological data, favor the continuity of the disease [6, 7].

The current treatment for Schistosomiasis is centered around praziquantel, an acylated quinoline-pyrazine derivative, that is able to act against the main strains of *S. mansoni* that infect humans and has been used for >30 years [8]. However, the exclusive use of praziquantel has been accompanied with reports in the literature that some patients have been resistant to pharmacological treatment [9-11]. Besides, praziquantel has no effect on immature schistosomes and cannot prevent reinfection or alter the schistosome life cycle [12, 13]. If it is considered that there is no effective alternative for the control of schistosomiasis, it is evident that the risk of developing resistance to praziquantel will increase in the coming decades, being a concern for the medical community. In view of this scenario, research priorities for facing schistosomiasis include the development and test of new drugs, test combinations of praziquantel with new drugs as well as monitoring the spread of praziquantel drug resistance [1].

In view of the need to obtain alternatives for the treatment of schistosomiasis and other neglected diseases, efforts have been made within the medicinal chemistry, using various planning techniques to obtain substances with improved structural characteristics and with less toxicity [14, 15] . These attempts comprehend praziquantel chemical structure modification by synthesis and evaluation of its analogues, rational design of new pharmacophores and discovery of new active compounds from screening programs on a large scale [16].

A class of derivatives that has gained attention because of its promising experimental anti-schistosomal activity is the one bearing the thiazole nucleus, which is effective against both immature and adult *Schistosoma mansoni* worms [17]. The thiazole moiety plays an important role in the medicinal chemistry field since it serves as scaffold for new drug synthesis, being already used for example as antimicrobial (sulfathiazole), anthelmintic and fungicide (thiabenzadole) and schistosomicide (niridazole) compounds [18-20]. Niridazole (Ambilhar®) started being used for treatment in 1964, but patients had little tolerance to this drug with serious side effects due to the presence of nitroaromatic chemical groups that act as parasitophores (their presence is essential to kill the worm) and also cause damage to host DNA by formation of adducts that induce mutagenesis [16]. However, advances in the development of medicinal chemistry tools have recently allowed the synthesis of new thiazole derivatives with anti-schistosomal activity. As an example, Santiago, de Oliveira (21) synthetized a set of molecules whose structures have a hydrazine and/or thiazole nucleus as a common group, and different pharmacophores including thiosemicarbazone, phthalyl thiosemicarbazone, phthalyl thiazole, and phthalyl thiazolidinone, and among them the compounds comprised of thiazole and phthalimide were the ones that exhibited the highest activity against worms, with a significant decline in motility, pairing and oviposition, as well as a mortality rate of 100% after 144 h of treatment.

Recently, Reddy, Kumar (22) described the synthesis of a library of hydrazinyl-thiazoles via a three-component reaction of various aldehydes/ketones with thiosemicarbazide and different phenacyl bromides, but the authors did not evaluate any biological potential of the derivatives. Based on the potentially promising features of these new compounds, here we selected eight aryl-thiazole derivatives and examined their efficacy in terms of (i) schistosome survival, (ii) egg output (oviposition), (iii) motility, (iv) couples pairing, (v) ultrastructural alterations in the tegument of *S. mansoni* as determined by scanning electron microscopy (SEM), and (v) expression of *S. mansoni* developmental genes.

MATERIALS AND METHODS

Chemistry

Synthesis of the compounds bioassayed in this study was previously described [22-24]. Production of aryl-thiazole derivatives was carried out at the Laboratory of Chemistry and Therapeutic Innovation, at the Federal University of Pernambuco. Synthesis was carried out in two steps (Fig 1).

Fig 1. Route of synthesis to obtain the aryl-thiazole derivatives (NJ series). In the compounds, R is hydrogen, 4-chloro, 4-bromo or 4-nitro. For 2,4-dimethoxy-aryl thiazole (right side route) R=Cl for NJ03; R=NO_2 for NJ06; R=Br for NJ08 and R=H for NJ12. For 3,4,5-trimethoxy-aryl thiazole (left side route) R=Cl for NJ04; R=NO_2 for NJ05; R=Br for NJ07 and R=H for NJ11.

In the first step, the thiosemicarbazide reacted in equimolar amounts with 2,4-dimethoxy-benzaldehyde (right side route) or 3,4,5-trimethoxy-benzaldehyde (left side route) through a condensation reaction in the presence of hydrochloric acid as the reaction catalyst to obtain a thiosemicarbazone (TSC), which served as an intermediary for thiazole synthesis following its acquisition. This second step was carried out in the presence of different 2-chloro or 2-bromoacetophenones (unsubstituted, 4-chloro-phenyl, 4-bromo-phenyl, 4-nitro-phenyl) under basic conditions (excess sodium acetate) and reflux [25].

Reactions were monitored with analytical thin-layer chromatography in silica gel 60 F254 plates and visualized under UV light (254 nm). Melting points were determined on a Quimis 340 capillary melting point apparatus and were not corrected. IR spectra were measured on Bruker IFS-66 IR spectrophotometer (Bruker, Germany) using KBr pellets. NMR spectra were recorded on Bruker AMX-300 MHz (300 MHz for ^{1}H and 75 MHz for ^{13}C) instruments by using tetramethylsilane as an internal standard. The spectroscopic data of the synthesized compounds (NJ03 to NJ08, NJ11 and NJ12) are in accordance with the structures proposed in Fig 1 and with the spectra published by Reddy, Kumar (22).

Ethics statement

The experimental protocols were in accordance with the Ethical Principles in Animal Research adopted by the Brazilian College of Animal Experimentation (COBEA) and the protocol/experiments have been approved by the Ethics Committee for Animal Experimentation of Institute Butantan (CEUA Nº 1777050816).

Maintenance of parasite life-cycle

The BH strain of *Schistosoma mansoni* (Belo Horizonte, Brazil) was maintained in the intermediate snail host *Biomphalaria glabrata* and the golden hamster (*Mesocricetus auratus*) was used as definitive host. Female hamsters aged 3-4 weeks, freshly weaned, weighing 50-60 g, were housed in cages (30x20x13cm) containing a sterile bed of wood shavings. A standard diet (Nuvilab CR-1 Irradiada, Quimtia S/A, Paraná, Brazil) and water were made available *ad libitum*. The room temperature was kept at 22 ± 2°C and a 12:12 hour light–dark cycle was maintained. Hamsters were infected by exposure to a *S. mansoni* cercariae suspension containing approximately 200-250 cercariae using the ring technique [26]. After 49 days of infection, the *S. mansoni* adult worms were recovered by perfusion of the hepatic portal system [27]. Cercariae were released from infected snails and mechanically transformed to obtain schistosomula *in vitro* [28].

Treatment of adult worms with NJ series compounds

Schistosomula and adult worms were treated with different concentrations of compounds from the NJ series in culture medium specific to each stage as indicated below (from a stock solution of 20 mM NJ series compound in dimethyl sulfoxide – (DMSO), and with the equivalent amount of DMSO in the control assays. Newly transformed schistosomula (NTS) were maintained for 2 h in M169 (Vitrocell) medium supplemented with 2% fetal bovine serum (FBS) (Vitrocell), 1 μM serotonin, 0.5 μM hypoxanthine,1 μM hydrocortisone, 0.2 μM triiodothyronine, penicillin/streptomycin, amphotericin, gentamicin (Vitrocell) at 37°C and 5% CO_2 [29]. Only after 2h incubation in culture medium the NJ series treatment was initiated. Paired adult worms freshly perfused from infected hamsters (see above) were maintained in culture in RPMI medium (Gibco) supplemented with 10% fetal bovine serum (FBS) (Vitrocell), penicillin/streptomycin, amphotericin,

gentamicin (Vitrocell) at 37°C and 5% CO_2 for two hours prior to the beginning of treatment with NJ series compound in DMSO or with 0.1% DMSO alone (control), which were added to the culture.

Motility assay

To evaluate the general condition of adult worms, including motility and mortality rate an inverted microscope was used. Parasites were observed after 1, 2 and 3 days of treatment with different concentrations of NJ series compounds or vehicle dimethyl sulfoxide (DMSO 0.1%). Motility and survival of worms were assessed according to the criteria scored in a viability scale of 0–3 [30]. The scoring system was as follows: 3, complete body movement; 1.5, partial body movement or immobile but alive; and 0, dead. Treatment was considered lethal whenever no worm movement was detected when observed for 2 min. Oviposition and ~~pairing status~~ were also observed and eggs were counted.

Viability assay

Viability of schistosomula and *S. mansoni* adult worms after treatment was determined by a cytotoxicity assay based on the CellTiter-Glo® Luminescent Cell Viability Assay kit (G7570, Promega, Madison, Wisconsin, EUA) [31]. The assay determines the amount of ATP present in freshly lysed adults or in intact schistosomula; the assay signals the presence of metabolically active cells.

In addition, viability of schistosomula was evaluated by the presence or absence of dead parasites, through staining with propidium iodide (PI) [32]. Schistosomula were equally distributed in 96-well microtiter plates, incubated with the different concentrations of NJ05 + NJ07 compounds indicated in the figure or the corresponding DMSO vehicle (control), and 2 µg/mL propidium iodide (PI) (Sigma-Aldrich) were added at 2 days of drug exposure. The parasites were immediately observed with light microscopy at 10 x magnification using a Nikon Eclipse fluorescent inverted microscope. Under fluorescence microscopy, schistosomula death was scored by a red fluorescence signal (572 nm emission microscope filter) [32, 33]. The number of biological replicates that were assayed, as well as the number of parasites that were counted per replicate, is stated in the legends to the figures.

Scanning electron microscopy (SEM)

Adult worms collected by perfusion were immediately transferred to supplemented RPMI medium; parasites were distributed in 6-well plates (adults: 10 paired worm couples per well) with medium. The worms were kept in culture (oven at 37 °C and 5 % CO_2) for 2 h for adaptation, and then NJ series compounds or the equivalent amount of 0.1% DMSO vehicle were added.

~~Ultrastructural analysis was performed with scanning electron microscopy.~~ Adult worms incubated at different concentrations of NJ series compounds or with 0.1% DMSO vehicle for 1 and 2 days were fixed with modified Karnovsky reagent (1% paraformaldehyde, 2.5 % glutaraldehyde, 1 mM calcium chloride in 1 M sodium cacodylate buffer, pH 7.4) and after the fixing stage the material was washed with sodium cacodylate buffer (0.1 mol / L, pH 7.2) and post-fixed with 1 % osmium tetroxide (w / v) for 1 h.

Samples were dehydrated with increasing concentrations of ethanol and then dried with liquid CO_2 in a critical-point dryer machine (model Leica EM CPD030, Leica Microsystems, Illinois, USA). Treated specimens were mounted on aluminum microscopy stubs and coated with gold particles using an ion-sputtering apparatus (model Leica EM SCD050, Leica Microsystems, Illinois, USA) [34]. Specimens were then observed and photographed using an electron microscope (FEI QUANTA 250, *Thermo Fisher Scientific, Oregon,* USA).

RT-qPCR validation of *S. mansoni* developmental genes

For quantitative RT-PCR, complementary DNAs were obtained by reverse transcription (RT) of 50 ng adult worms total RNA using SuperScript IV Reverse Transcriptase (Invitrogen) and random hexamer primers in a 20 µL volume, according to the manufacturer's recommendations. The resulting cDNA was diluted 8-fold in water and qPCR amplification was done with 2.5 µL of diluted cDNA in a total volume of 10 µL using SYBR Green Master Mix (Life Technologies) and specific primer pairs (Table S1). Tested genes were described in previous publications from the literature [35-40] or selected here based on their predicted function, and primers were designed with the use of Primer3 online software. The Light cycle 480 II (Roche) qPCR was used. Results were analyzed by comparative Ct method and the statistical significance was calculated with the two-sided t-test.

To find adequate normalizer genes for qPCR, we looked for evidence of genes with non-detectable changes in expression upon NJ series compounds treatment; ten genes were selected based on literature reports [35-40] and tested by qPCR in the adult female worms for the lack of effect of NJ series compounds on their levels of expression. The two least affected genes were Mitochondrial 28s ribosomal protein s14 (Smp_090920) and PI3K regulatory subunit 4 (Smp_123610). In all cases, the geometric mean expression of these two genes was used as normalizer for calculating the expression levels of genes of interest.

RESULTS

Phenotypic effects of NJ series compounds on *S. mansoni* schistosomula

Eight dimethoxy-aryl thiazole derivatives (Fig 1) were tested for schistosomicidal properties against the schistosomula early forms of the parasite. For this, schistosomula mechanically transformed from cercariae were pre-incubated for 2 h in culture medium, each compound indicated in Fig 2A and Figure S1 was subsequently added, and their effect on schistosomula viability was evaluated every day for a period of five days by measuring the concentration of ATP in the metabolically active cells of the parasite. Different concentrations of each compound from 6.25 to 50 µM were tested, and out of the eight compounds that were assayed (Fig 2A and S1 Text, Fig A), two had a more prominent effect in reducing schistosomula viability, namely NJ05 and NJ07 (Fig 2A). These compounds after five days of exposure decreased the parasites' viability by approximately 40 % (Fig 2A). In order to test for a possible synergistic effect, the best two compounds were used together for schistosomula treatment. Within the first day of treatment with NJ05 + NJ07, across all concentrations tested (12.5, 25 and 50 µM) there was a dose-dependent decrease of 60 to 80% in parasite viability (Fig 2B), indicating a clear synergy between the two compounds. The phenotype was more prominent after five days of treatment, with viability reduced to zero (Fig 2B). The effect on schistosomula viability over 5 days of exposure to each of the other six NJ series compounds is shown in S1 Text Fig A (see Supporting Information); a less marked 10 to 40 % reduction in parasites viability was observed.

Fig 2. Synergistic effect of NJ05 + NJ07 on schistosomula viability. (A and B) ATP quantitation using a luminescent assay to assess schistosomula viability under NJ series compounds exposure. Schistosomula (100-120/well) were incubated with the indicated concentrations of NJ05, NJ07, NJ05 + NJ07 or with vehicle (0.1% DMSO) for up to 5 days. Viability was expressed as % luminescence values relative to the control (DMSO). Mean ± SEM from three replicate experiments. The two-way ANOVA test was used to calculate the statistical significance (*p < 0.05; **p < 0.01; ***p < 0.001). For clarity purposes, we show only the highest p-value obtained from the two-way ANOVA test for each time point on all different concentrations tested.

(C) Quantitation of schistosomula viability using propidium iodide staining; schistosomula were treated for two days with NJ05 + NJ07 at the different concentrations indicated. Percentage of viable schistosomula (non-stained with propidium iodide) is shown. For each condition tested, about 400 schistosomula were used, divided into four biological replicates. Mean ± SEM from four replicate experiments. **(D)** Schistosomula treated with the indicated concentrations of NJ05 + NJ07 or with vehicle (0.1% DMSO) for 2 days were visualized by staining with propidium iodide (marker of dead cells; 572 nm emission filter microscope). For each concentration (indicated at the top), the upper panel shows a light microscopy image and the bottom panel shows the image of the same field with differential fluorescence detection of PI-positive parasites. *Bar= 100 µm.*

Propidium iodide (PI) was used as an alternate method to quantitate viability of schistosomula after a 2-days incubation in the presence of control vehicle (0.1% DMSO) or of 6.25 to 50 µM of the best two compounds together (NJ05 + NJ07) (Fig 2C and 2D). Under fluorescence microscopy, dead schistosomula were detected by a red fluorescence signal from PI (536 nm emission microscope filter) (Fig 2D, lower panels). Total number of schistosomula present in the field was detected and counted under light optical microscopy at each NJ05 + NJ07 concentration (Fig. 2D, upper panels), and the percentage viability was computed and plotted as shown in Fig 2C; mortality was 100 % with 25 and 50 µM NJ05 + NJ07. Incubation with control vehicle (0.1 % DMSO) for 2 days showed no reduction in parasite viability (Fig 2B and Fig 2D, first upper and lower panels).

Effects of NJ series compounds on *S. mansoni* adult worms' viability and motility

In order to evaluate the schistosomicidal activity of all eight compounds on adult worms we measured oviposition, ~~male-female pairing status,~~ worms' viability, motility, and alterations in the tegument. Compounds were tested at a concentration range of 12.5 to 50 µM and observed every day for a period of 3 days.

Again, compounds NJ05, NJ07 and NJ05 + NJ07 exhibited the highest schistosomicidal activities among the eight tested, showing a concentration-dependent significant decrease in

viability of the adult worms, as evaluated by ATP quantitation (Fig 3A – 3C). Compound NJ05 was more effective than NJ07, and viability after three days was reduced to 25 % in parasites treated with 50 µM NJ05 (Fig 3A), compared with a viability reduction to 40 % with 50 µM NJ07 (Fig 3B). No synergistic effect was detected when the two compounds were tested together (NJ05 + NJ07), as the viability was reduced to approximately 25 % after three days of exposure to 50 µM NJ05 + NJ07 (Fig 3C), similar to the reduction caused by NJ05 only. The effect of other tested compounds is shown in S1 Text Fig B (see Supporting Information); after three days of treatment with compounds NJ03, NJ04 and NJ08 (at 50 µM concentration), a reduction in viability to only 75 % was observed.

Fig 3. *In vitro* effects of NJ05, NJ07 and NJ05 + NJ07 on the viability and motility of *Schistosoma mansoni* adult worms. (A - C) Viability was estimated by the total amount of ATP available in the parasites, using a luminescent assay. Pairs of adult worms were treated for 2 days with NJ05, NJ07 and NJ05 + NJ07 at the different concentrations indicated or with vehicle (0.1% DMSO). Viability was expressed as percentage luminescence values relative to the control (DMSO). Mean ± SEM from three replicate experiments, each with 10 worm pairs. **(D - F)** Percentage of relative motility of adult worms treated with different concentrations of NJ05, NJ07 and NJ05 + NJ07 and controls (0.1 % DMSO) at different times of exposure from 1 to 3 days. Mean ± SEM of three experiments, each with 10 worm pairs. *$p < 0.05$, **$p < 0.01$ and *** $p < 0.001$ compared with controls. For clarity purposes, we show only the highest p-value obtained from the two-way ANOVA test for each time point on all different concentrations tested.

S. mansoni adult worms exhibited decreased motility when exposed to NJ05, NJ07 and NJ05 + NJ07, and this was dependent upon concentration and incubation time (Fig 3D – 3F). After three days with 50 µM NJ05, NJ07 or NJ05 + NJ07 motility decreased to 30 – 50 % compared with controls (Fig 3D – 3F). Impairment of the peristaltic movement and reduced ability of the suckers to adhere to the bottom of the culture plates were observed. Among the other compounds of the NJ series tested we can highlight the 50 % decrease in motility of adult worms exposed to 50 µM NJ03 or NJ08 after three days of exposure (S1 Text Fig C; see Supporting Information).

Effects of NJ series compounds on *S. mansoni* adult worm couples pairing and female oviposition

Compound NJ05 showed a more pronounced effect than NJ07 on worm couples pairing (Fig 4A and 4B). Thus, couples exposed for one day to 25 μM or 50 μM NJ05 already exhibited no pairing (Fig 4A), whereas at day one with 25 μM NJ07, couple pairing was not reduced and with 50 μM NJ07, pairing was reduced to approximately 60 % compared with control (Fig 4B). Combination of NJ05 + NJ07 was not effective in enhancing couples unpairing (Fig 4C) when compared with NJ05 alone. Among the other compounds of the NJ series reported in S1 Text Fig D (see Supporting Information), we can highlight the unpairing of approximately 75 % - 90 % of adult worm couples after three days exposure to 50 μM NJ03, NJ04, NJ08 or NJ11.

Fig 4. *In vitro* effects of NJ05, NJ07 and NJ05 + NJ07 on the pairing and oviposition of *Schistosoma mansoni* adult worms. (A-C) Monitoring the pairing of control couples and of couples treated with different concentrations of NJ05, NJ07 and NJ05 + NJ07 at different times of exposure from 1 to 3 days. Mean ± SEM of three experiments, each with 10 worm pairs. **(D-F)** Number of eggs released by females incubated with NJ05, NJ07 and NJ05 + NJ07 at different concentrations or with vehicle (0.1 % DMSO) at different times of exposure from 1 to 3 days. *p < 0.05, **p < 0.01 and *** p < 0.001. For clarity purposes, we show only the highest p-value obtained from the two-way ANOVA test for each time point on all different concentrations tested.

In addition, oviposition was extremely affected by treatment with the NJ series compounds, especially NJ05, NJ07 and NJ05 + NJ07 (Fig 4D – 4F). Of note, reduction in oviposition was independent from couple unpairing. Thus, at concentrations of 25 μM and 50 μM, oviposition reached almost zero with NJ05, NJ07 and NJ05 + NJ07 already at day one (Fig 4D – 4F), even when unpairing was incomplete or not present such as with NJ07 (compare Fig 4B and 4E). Results for reduction in oviposition of the other tested compounds are described in S1 Text Fig E (see Supporting Information); little effect was observed in oviposition with these other compounds.

Scanning electron microscopy of treated adult worms

Through scanning electron microscopy (SEM) it was possible to detect phenotypic alterations between control and treated adult worms. Control adult male schistosomes that were not exposed to any drugs are depicted in Fig 5 (panels 1 – 4) and Fig 6 (panels 1 – 4), showing a normal topography of the surface membrane of anterior and dorsal regions, respectively. Oral and ventral suckers can be clearly visualized (Fig 5, panels 2 – 4). In the anterior portion of the body, the gynecophoral canal, a longitudinal fold of the middle and posterior body that houses the female for the purpose of mating and reproduction, can be identified (Fig 5, panel 2). In Fig 6, panels 1 to 4, the medial and posterior parts of schistosomes are shown, respectively. In these regions, a large number of tubercles with typical spines and ciliated papillae was observed (Fig 6, panels 2 and 3).

Fig 5. Micrographs showing alterations on the ultrastructure of the anterior region of *Schistosoma mansoni* adult male worms exposed to NJ05 or NJ07. Scanning electron microscopy of worms exposed for 2 days to 25 μM NJ05 (panels 5 – 8), 25 μM NJ07 (panels 9 – 12) or vehicle (0.1% DMSO) (panels 1 – 4). Note that the size scale bar is shown within the black thin line below each image; **panels 1 and 2**: Control worms (Bar = 500 μm; 100 μm) presenting the fully paired couple; **panels 3 and 4**: Anterior region of control male presenting normal oral and ventral suckers tegument (Bar = 50 μm; 30 μm); **panels 5 and 6**: Anterior region of adult male worm treated with 25 μM NJ05 (Bar = 500 μm; 100 μm); **panels 7 and 8**: Male oral and ventral suckers presenting tegument peeling (Bar = 50 μm); **panels 9 and 10:** Anterior region of male adult worm treated with 25 μM NJ07 (Bar = 500 μm; 100 μm); **panels 11 and 12**: Male oral and ventral suckers presenting tegument without structural alterations (Bar = 50 μm). *f*: *female; os*: *oral sucker; vs*: *ventral sucker; gc: gynecophoral canal*; *la*: *lesion area; cp: ciliated papillae*.

Fig 6. Scanning electron micrographs of dorsal and posterior regions of *Schistosoma mansoni* adult male worm exposed to NJ05 or NJ07. Worms were exposed for 2 days to 25 μM NJ05 (panels 5 – 8), 25 μM NJ07 (panels 9 – 12) or vehicle (0.1% DMSO) (panels 1 – 4). **Panels 1 to 4**: Medial (panels 1 – 3) and posterior (panel 4) regions of control male worm presenting normal tegument (Bar = 50 μm; 20 μm; 5 μm; 50 μm). Note that the size scale bar is shown within the black thin line below each image. **Panels 5 to 7**: Enlarged view of dorsal region of adult male worm

treated with NJ05 showing tegument lesions and loss of tubercles (Bar = 50 μm; 20 μm; 5 μm; 50 μm); **panel 8**: Posterior region of male adult worm treated with NJ05 showing lesion areas (Bar = 50 μm); **panels 9 to 11**: Dorsal region of male adult worm treated with NJ07 showing bubbles throughout (Bar = 30 μm; 20 μm; 5 μm; 50 μm); **panel 12**: Posterior region of male adult worm treated with NJ07 (Bar = 50 μm). ~~*cp: ciliated papillae*~~*; b: blebs; tb*: *tubercles; la*: *lesion area*.

SEM revealed detailed surface membrane ultrastructural damage on the anterior (Fig 5) and dorsal (Fig 6) regions of adult male worms caused by *in vitro* exposure for 2 days to 25 μM NJ05 (Fig 5 and 6, panels 5 – 8) and NJ07 (Fig 5 and 6, panels 9 – 12) compared with controls (0.1% DMSO) (Figs 5, and 6, panels 1 – 4). Thus, incubation of adult male *S. mansoni* worms with NJ05 resulted in severe tegument destruction of the anterior region (Fig 5, panels 5 – 8). In the ventral anterior region, focal lesions were observed in the oral and ventral suckers (Fig 5, panels 7 and 8). In the anterior portion of the body exposed to NJ07 the tegument showed no alterations (Fig 5, panels 9 – 12), and exhibited numerous ciliated papillae similar to the control (Fig 5, panels 1 – 4).

Severe damage to the dorsal surface of adult male schistosomes was induced by incubation for 2 days with NJ05 (Fig 6, panels 5 – 8) or NJ07 (Fig 6, panels 9 – 12). With NJ05, deterioration of the dorsal tegument was observed at higher magnification (Fig 6, panels 5 – 7), with the presence of lesion areas and loss of numerous tubercles and spines that covered the whole body of the parasite. In the posterior region, focal lesions along the body became evident (Fig 6, panel 8). Countless bubbles and loss of ciliated papillae were observed in the dorsal region of male worm exposed to NJ07 (Fig 6, panels 9 – 11); tegument at the male worm posterior region had no structural alterations (Fig 6, panel 12). Similar structural changes were already detected in adult male worms exposed for only 24 h to NJ05 or NJ07, with a more pronounced effect of NJ05 (S1 Text Fig F, see Supporting Information).

Control females incubated with vehicle (DMSO 0.1%) showed a normal surface (Fig 7, panels 1– 4). In females treated with 25 μM NJ05 for 2 days, severe damage to the dorsal tegument was visualized (Fig 7, panels 6 – 8), exhibiting extensive sloughing that exposed

subtegumental tissues. Presence of blisters can be seen throughout the extension of the dorsal region of female exposed to 25 µM NJ07 for 2 days (Fig 7, panels 10 – 12).

Fig 7. Damaged dorsal surface of *S. mansoni* adult female worms exposed to NJ05 or NJ07. Female worms were exposed for 2 days to 25 µM NJ05 (panels 5 – 8), 25 µM NJ07 (panels 9 – 12) or to vehicle (DMSO 0.1%) (panels 1 – 4) and were observed by scanning electron microscopy; note that the size scale bar is shown within the black thin line below each image. **panel 1:** Low magnification image of female control worm (Bar = 500 µm). **panels 2 to 4:** Images of female control worms dorsal region, with normal structural appearance (Bar = 40 µm; 10 µm and 4 µm); **panel 5:** Low magnification image of female worm exposed to NJ05 (Bar = 500 µm); **panels 6 to 8:** Middle dorsal region of female presenting apparent surface damage with peeling of the tegument membrane (Bar = 50 µm; 10 µm and 5 µm); **panel 9:** Low magnification image of female worm exposed to NJ07 (Bar = 500 µm); **panels 10 to 12:** Dorsal region of female worm presenting bubbles along the tegument (Bar = 30 µm; 10 µm and 4 µm). *p: peeling; la: lesion area; b: blebs.*

Assessment by quantitative PCR of the expression of genes involved with vitellaria and female reproduction

A set of six genes that are involved in egg formation, and participate in cell differentiation was selected for assessment by RT-qPCR of their possible change in expression caused by NJ series compounds. Tested genes involved in egg biosynthesis were all confirmed by qPCR as downregulated in females treated with 25 µM NJ05 for 2 days (Fig 8), with a significant reduction in expression of p14, Tyrosinase 2, p48 and fs800. Two other genes tested by qPCR showed a tendency to be less expressed in treated females (Fig 8), although exhibiting non-statistically-significant changes; they were the Egg Shell Protein (ESP) gene, and the nanos gene, which is involved in vitelline cell differentiation [41]. It is evident that NJ05 had a direct effect on the expression level of selected genes that are involved with eggshell formation and egg development in *S. mansoni* females.

The effect of NJ07 or NJ05 + NJ07 on the expression of the same set of six genes was also tested; worms were treated for 2 days with 25 μM NJ07 or NJ05 + NJ07; the only gene detected by RT-qPCR as significantly 2.8 – 2.9 X downregulated in females under both treatments was nanos, the gene related to cell differentiation (S1 Text Fig G, see Supporting Information). Although oviposition was impaired in both treatment conditions (see Fig 4E and 4F), there was no significant change in expression caused by both treatments among the five tested genes related to eggshell formation and egg development (S1 Text Fig G, see Supporting Information).

Fig 8. Vitellaria and female reproduction gene expression inhibition in adult females upon NJ05 treatment. Adult couples were treated with NJ05 (25 μM) or with vehicle DMSO (0.1 %) for 2 days. The parasites were stored in RNAlater for further processing. All parasite couples were separated and only the females had their RNA extracted followed by cDNA synthesis. The genes measured by qPCR were Smp_131110 (p14), Smp_000430 (Egg Shell Protein (ESP)), Smp_013540 (Tyrosinase 2 (Tyr 2)), Smp_014610 (p48), Smp_000290 (fs800), Smp_055740 (Nanos 2). The geometric mean from two reference genes was used for normalization with the DCT method (Smp_090920 and Smp_123610). The plotted data is retrieved from DDCT analyses in which the DMSO-treated sample is the control. Significant fold-change in gene expression between DMSO and NJ05 treatment is shown by the numbers inside the brackets. Mean ± SEM of four replicates is shown. Student unpaired parametric two-sided t-test was used and statistically significant differences are represented by the asterisks. *p≤0.05; **p≤0.01.

DISCUSSION

Chemotherapy is the only immediate recourse for minimizing the prevalence of schistosomiasis, and currently it involves predominately the administration of a single drug, praziquantel (PZQ). There is an important limitation of the therapeutic profile of PZQ, as it lacks activity against larval developing stages of the parasite; therefore, retreatment is necessary to kill those parasites that have since matured [21, 42]. The present study was performed to evaluate the *in vitro* schistosomicidal effect of the aryl-thiazole derivatives both on adults and ~~on the larval developing stages of the parasite.~~ The following schistosomicidal parameters were analyzed: viability, motility, oviposition, mortality and morphological changes, which are indicators commonly

used for evaluating the schistosomicidal effect of new chemical entities [43, 44]. In light of the parasite biology and according to the evaluation of these parameters, schistosomicidal activity can be effective essentially in three different ways: causing schistosomula death, thus preventing host infection, inhibiting oviposition that could lead to a decrease in the pathological phenotype of schistosomiasis (hepato- and splenomegaly), and causing adult worm's death [44, 45]. All three of the above schistosomicidal effects were present in our *in vitro* assays with NJ compounds, suggesting that further exploration of this class of aryl-thiazole derivatives is warranted.

The aryl-thiazole compounds evaluated in this study are differentiated, in principle, by the substitution pattern of the phenyl ring directly attached to the hydrazine portion (2,4-dimethoxy-phenyl and 3,4,5-trimethoxy-phenyl). Trimethoxylated derivatives (NJ04, NJ05, NJ07 and NJ11) showed a better profile in reducing the viability of *S. mansoni* schistosomula and adult worms. The main electronic change on the molecule caused by tri-substitution is an increase of the electronic density, and this effect significantly increased the schistosomicidal activity, also demonstrated by the synergic effect of compounds NJ05 + NJ07 which differ only by 4-nitro and 4-bromo substituents, respectively. The results corroborate with studies involving other parasites such as *Plasmodium falciparum* [46], *Leishmania braziliensis* [47] and *Trypanosoma cruzi* [48], evidencing the importance of methoxy group trisubstituted aromatic nuclei for antiparasitic activity.

Additionally, the two aryl-thiazole series are discriminated by the presence of different substituents of the phenyl ring attached to the thiazole nucleus (hydrogen, 4-chloro, 4-bromo, 4-nitro). The best compounds evaluated in this study have 4-nitro (NJ05) and 4-bromo (NJ07). Notably, such substitutions reflect an increase in antiparasitic activity as described in the literature. Papadopoulou, Bloomer (49) described the antitrypanosomal activity of 5-nitro-2-aminothiazole and demonstrated activity against *T. cruzi* amastigotes at nM concentrations and was about 4-fold more potent than BNZ (benznidazole-reference drug). Generally, the biological activity of nitro compounds involves the biotransformation of the nitro group, releasing intermediates that disrupt the redox process. Some of those intermediates can attack enzymes, membranes and DNA, providing the basis for their mode of action, including those reported against parasites [50]. NJ compounds might have caused oxidative stress and the consequent biological activity observed here. It is worthwhile to highlight that direct amino acid nitrosylation of SmTGR (Thioredoxin-

Glutathione Reductase) enzyme has been reported for recombinant *S. mansoni* TGR after reaction with furoxan [51], demonstrating the potential of compounds bearing nitro moieties since the SmTGR enzyme is essential for parasite survival and has been the subject of intense studies for identification of potential schistosomicidal agents [51].

The insertion of halogen atoms, in turn, on the compound structure in the discovery phase of new drugs can lead to increased potency and selectivity of the pharmacological target by affecting the pKa, modulating the conformation, increasing hydrophobic interactions between the ligand and the target, increasing lipophilicity and favoring permeation in biological membranes [52, 53]. The increase in cellular permeability of the parasite membrane is an important feature for the action of a target drug; for example, PZQ is supposed to act on the voltage-gated calcium channels of schistosomes [54], but has also been shown to alter membrane fluidity in model membranes [55] and in the *S. mansoni* tegument [56], thus possibly increasing calcium permeability. Permeation in biological membranes may have been fundamental for the good activity presented by NJ07. The phenotypic effects observed over the course of NJ05 treatment on *S. mansoni* couples included reduction in motility, impairment of peristaltic movement and of the ability of their suction suckers to adhere to the bottom of the culture plates, thus generating weak contractions and subsequent paralysis. This may be a consequence of the perturbation in the influx of extracellular calcium, which in eukaryotic cells is a signal for motor events, besides playing an important role in the promotion of apoptosis [57-59].

Changes in the general conditions of worms, including adult worms' motility and tegument damage visualized through high resolution scanning electron microscopy were observed after two days treatment with 25 μM NJ05 + NJ07. Motility assay and cytotoxicity evaluation by ATP quantification revealed an increase in the number of dead parasites that was concentration and time dependent following NJ05 + NJ07 treatment. Maximum death of adult worms occurred after three days of treatment at a concentration of 25 or 50 μM NJ05 + NJ07. Noteworthy, in schistosomula 100 % death happened in the first two days (25 or 50 μM NJ05 + NJ07). The larvae presented lack of movement, granularity and shape alterations, showing that NJ05 + NJ07 combination was effective against ~~younger~~ forms of the parasite. This is of great importance, since

the drugs of choice for treatment of schistosomiasis act on adult worms, having a small action on schistosomula [60].

Detailed microscopic observation showed that NJ05 and NJ07 were capable, separately, of inducing morphological alterations in the outer membrane of all adult worms tested in this work. We found extensive damage to the tegument in both male and female worms after 2 days of exposure of NJ05 and NJ07. Male adult worms of *S. mansoni* treated with NJ05 showed lesions of tegument and loss of tubercles, the females, in turn, demonstrated severe damage on the tegument surface with the presence of blisters and large peeling of the tegument. With NJ07 the dorsal region of male and female adult worms showed bubbles in all their extension. Alterations in the surface ultrastructure of schistosome worms have been investigated by a number of authors in order to evaluate anti-schistosomal drugs [61-66] . Here, the alterations caused by NJ05 and NJ07 indicate that trimethoxy substituent and the presence of NO_2 and Br substituent favor the damage on male and female adult worms.

The compounds tested here appear to influence adult females' oviposition, mainly NJ05 and NJ05 + NJ07, since no eggs at all were found in the culture medium after treatment. Eggs obtained from couples treated with NJ07 were small and not viable. In females treated with NJ05, the expression of genes related to egg biosynthesis including tyrosinase (SmTyr2) [67], eggshell precursors such as p14 [68] and p48 [69] was found to be decreased in relation to the control.

Also, genes related to cell differentiation and development of the germinative line of metazoans such as Nanos were found to have decreased expression in relation to the control in adult females treated with NJ05, NJ07 and NJ05 + NJ07. On platyhelminths, Nanos is generally found to be expressed in the anterior portion of the ovary [41], where the oogonias are located [70], and it plays an essential role in the proper development, regeneration and maintenance of germ cells in several organisms [71-73].

Overall, the results indicate that thiazoles, especially those trimethoxylated derivatives are antiparasitic agents. These compounds showed substantial schistosomicidal properties against schistosomula and adult *S. mansoni* worms, with a significant reduction in viability, motility, pairing, survival rate, oviposition, severe alterations in the tegument and mortality of worms, and further biological studies are warranted to clarify the mechanisms of schistosomicidal action. It is important

to emphasize that NJ05 + NJ07 had an intense activity against the young forms of Schistosoma, a characteristic not encountered in the drugs of choice for treatment of schistosomiasis such as PZQ and oxamniquine [8, 74]. The present study revealed NJ05 to possess the most effective schistosomicidal properties, suggesting its use as an aryl-thiazole prototype for the development of new schistosomicidal compounds.

Acknowledgments

We thank Beatriz Mauricio and Dr. Carlos Jared, Instituto Butantan, Sao Paulo, for access to the scanning electron microscopy facilities in their laboratories. We acknowledge Dr. Eliana Nakano and Patricia Aoki Miyasato, Instituto Butantan, Sao Paulo, and Dr. Pedro Luiz Silva Pinto, Instituto Adolfo Lutz, Sao Paulo, for maintaining the parasite life cycle.

References:

1. McManus DP, Dunne DW, Sacko M, Utzinger J, Vennervald BJ, Zhou XN. Schistosomiasis. Nature reviews Disease primers. 2018;4(1):13. Epub 2018/08/11. doi: 10.1038/s41572-018-0013-8. PubMed PMID: 30093684.

2. Gryseels B, Polman K, Clerinx J, Kestens L. Human schistosomiasis. Lancet. 2006;368(9541):1106-18. Epub 2006/09/26. doi: S0140-6736(06)69440-3 [pii]

10.1016/S0140-6736(06)69440-3. PubMed PMID: 16997665.

3. WHO. Schistosomiasis. Fact sheet: accessed 20 Feb 2019 2018.

4. Keiser J, Chollet J, Xiao SH, Mei JY, Jiao PY, Utzinger J, et al. Mefloquine--an aminoalcohol with promising antischistosomal properties in mice. PLoS Negl Trop Dis. 2009;3(1):e350. Epub 2009/01/07. doi: 10.1371/journal.pntd.0000350. PubMed PMID: 19125172; PubMed Central PMCID: PMC2600813.

5. Lescano SZ, Chieffi PP, Canhassi RR, Boulos M, Amato Neto V. [Antischistosomal activity of artemether in experimental Schistosomiasis mansoni]. Rev Saude Publica. 2004;38(1):71-5. Epub 2004/02/14. doi: S0034-89102004000100010 [pii]. PubMed PMID: 14963544.

6. Kuntz AN, Davioud-Charvet E, Sayed AA, Califf LL, Dessolin J, Arner ES, et al. Thioredoxin glutathione reductase from *Schistosoma mansoni:* an essential parasite enzyme and a key drug target. PLoS Med. 2007;4(6):e206. Epub 2007/06/21. doi: 06-PLME-RA-0587 [pii]

10.1371/journal.pmed.0040206. PubMed PMID: 17579510; PubMed Central PMCID: PMC1892040.

7. Katz N, Coelho PM. Clinical therapy of schistosomiasis mansoni: the Brazilian contribution. Acta Trop. 2008;108(2-3):72-8. Epub 2008/07/12. doi: 10.1016/j.actatropica.2008.05.006

S0001-706X(08)00141-1 [pii]. PubMed PMID: 18617155.

8. Siqueira LD, Fontes DAF, Aguilera CSB, Timoteo TRR, Angelos MA, Silva LCPBB, et al. Schistosomiasis: Drugs used and treatment strategies. Acta Tropica. 2017;176:179-87. doi: 10.1016/j.actatropica.2017.08.002. PubMed PMID: WOS:000413606800025.

9. Pica-Mattoccia L, Doenhoff MJ, Valle C, Basso A, Troiani AR, Liberti P, et al. Genetic analysis of decreased praziquantel sensitivity in a laboratory strain of *Schistosoma mansoni*. Acta Trop. 2009;111(1):82-5. Epub 2009/05/12. doi: 10.1016/j.actatropica.2009.01.012

S0001-706X(09)00030-8 [pii]. PubMed PMID: 19426668.

10. Melman SD, Steinauer ML, Cunningham C, Kubatko LS, Mwangi IN, Wynn NB, et al. Reduced susceptibility to praziquantel among naturally occurring Kenyan isolates of *Schistosoma mansoni*. PLoS Negl Trop Dis. 2009;3(8):e504. Epub 2009/08/19. doi: 10.1371/journal.pntd.0000504. PubMed PMID: 19688043; PubMed Central PMCID: PMC2721635.

11. Doenhoff MJ, Cioli D, Utzinger J. Praziquantel: mechanisms of action, resistance and new derivatives for schistosomiasis. Curr Opin Infect Dis. 2008;21(6):659-67. Epub 2008/11/04. doi: 10.1097/QCO.0b013e328318978f

00001432-200812000-00013 [pii]. PubMed PMID: 18978535.

12. Cioli D, Pica-Mattoccia L, Basso A, Guidi A. Schistosomiasis control: praziquantel forever? Mol Biochem Parasitol. 2014;195(1):23-9. Epub 2014/06/24. doi: 10.1016/j.molbiopara.2014.06.002. PubMed PMID: 24955523.

13. Ross AGP, Olveda RM, Li YS. An audacious goal: the elimination of schistosomiasis in our lifetime through mass drug administration. Lancet. 2015;385(9983):2220-1. doi: 10.1016/S0140-6736(14)61417-3. PubMed PMID: WOS:000355583000032.

14. Barreiro EJ, Fraga CAM, Miranda ALP, Rodrigues CR. A química medicinal de N-acilidrazonas: novos compostos-protótipos de fármacos analgésicos, antiinflamatórios e anti-trombóticos. Quím Nova. 2002;25(1):129-48. doi: http://dx.doi.org/10.1590/S0100-40422002000100022.

15. Manssour Fraga CA, Barreiro EJ. New Insights for Multifactorial Disease Therapy: The Challenge of the Symbiotic Drugs Current Drug Therapy. 2008;3(1):1-13. doi: https://doi.org/10.2174/157488508783331225.

16. da Silva VBR, Campos B, de Oliveira JF, Decout JL, do Carmo Alves de Lima M. Medicinal chemistry of antischistosomal drugs: Praziquantel and oxamniquine. Bioorg Med Chem. 2017;25(13):3259-77. Epub 2017/05/13. doi: 10.1016/j.bmc.2017.04.031. PubMed PMID: 28495384.

17. de Oliveira SA, de Oliveira Barbosa M, Filho C, Oliveira AR, de Sousa FA, de Farias Santiago E, et al. Phthalimido-thiazole as privileged scaffold: activity against immature and adult worms of *Schistosoma mansoni*. Parasitol Res. 2018;117(7):2105-15. Epub 2018/05/08. doi: 10.1007/s00436-018-5897-4. PubMed PMID: 29736731.

18. Kubba AARM, Abbas SS. Synthesis and antimicrobial evaluation of new derivatives derived from-2-amino-4-(4-nitro-/4-bromo-phenyl thiazole). Res J Pharm Biol Che. 2018;9(1):235-42. PubMed PMID: WOS:000428942500033.

19. Mohareb RM, Abdallah AEM, Ahmed EA. Synthesis and cytotoxicity evaluation of thiazole derivatives obtained from 2-amino-4,5,6,7-tetrahydrobenzo[b]thiophene- 3-carbonitrile. Acta Pharm. 2017;67(4):495-510. Epub 2018/01/18. doi: 10.1515/acph-2017-0040. PubMed PMID: 29337677.

20. Ouf SA, Gomha SM, Ewies MM, Sharawy IAA. Synthesis, Characterization, and Antifungal Activity Evaluation of Some Novel Arylazothiazoles. J Heterocyclic Chem. 2018;55(1):258-64. doi: 10.1002/jhet.3040. PubMed PMID: WOS:000422654400029.

21. Santiago EF, de Oliveira SA, de Oliveira Filho GB, Moreira DR, Gomes PA, da Silva AL, et al. Evaluation of the anti-*Schistosoma mansoni* activity of thiosemicarbazones and thiazoles. Antimicrobial agents and chemotherapy. 2014;58(1):352-63. Epub 2013/10/30. doi: 10.1128/AAC.01900-13. PubMed PMID: 24165185; PubMed Central PMCID: PMC3910798.

22. Reddy GT, Kumar G, Reddy NCG. Water-Mediated One-pot Three-Component Synthesis of Hydrazinyl-Thiazoles Catalyzed by Copper Oxide Nanoparticles Dispersed on Titanium Dioxide Support: A Green Catalytic Process. Adv Synth Catal. 2018;360(5):995-1006. doi: 10.1002/adsc.201701063. PubMed PMID: WOS:000426489300023.

23. Mazzone G, Pignatello R, Panico A, Mazzone S, Puglisi G, Pennisi G, et al. MAOI activity of some novel series of substituted thiazol-2-yl-hydrazines. Pharmazie. 1992;47(12):902-10. Epub 1992/12/01. PubMed PMID: 1293612.

24. Popova ZI, Tsurkan AA, Steblyuk PN. Synthesis and biological activity of some phenylazothiazoles. Farmatsevtichnii Zhurnal. 1985;6:41-4.

25. de Oliveira Filho GB, Cardoso MVO, Espindola JWP, Oliveira ESDA, Ferreira RS, Coelho PL, et al. Structural design, synthesis and pharmacological evaluation of thiazoles against *Trypanosoma cruzi*. Eur J Med Chem. 2017;141:346-61. Epub 2017/10/17. doi: S0223-5234(17)30762-6 [pii]

10.1016/j.ejmech.2017.09.047. PubMed PMID: 29031078.

26. Tucker MS, Karunaratne LB, Lewis FA, Freitas TC, Liang YS. Schistosomiasis. Current protocols in immunology. 2013;103:Unit 19 1. Epub 2014/02/11. doi: 10.1002/0471142735.im1901s103. PubMed PMID: 24510597.

27. Duvall RH, DeWitt WB. An improved perfusion technique for recovering adult schistosomes from laboratory animals. Am J Trop Med Hyg. 1967;16(4):483-6. Epub 1967/07/01. PubMed PMID: 4952149.

28. Ramalho-Pinto FJ, Gazzinelli G, Howells RE, Mota-Santos TA, Figueiredo EA, Pellegrino J. *Schistosoma mansoni*: defined system for stepwise transformation of cercaria to schistosomule in vitro. Exp Parasitol. 1974;36(3):360-72. Epub 1974/12/01. PubMed PMID: 4139038.

29. Basch PF. Cultivation of *Schistosoma mansoni* in vitro. I. Establishment of cultures from cercariae and development until pairing. J Parasitol. 1981;67(2):179-85. Epub 1981/04/01. PubMed PMID: 7241277.

30. Horiuchi A, Satou T, Akao N, Koike K, Fujita K, Nikaido T. The effect of free and polyethylene glycol-liposome-entrapped albendazole on larval mobility and number in Toxocara canis infected mice. Vet Parasitol. 2005;129(1-2):83-7. Epub 2005/04/09. doi: S0304-4017(05)00006-3 [pii]

10.1016/j.vetpar.2004.12.017. PubMed PMID: 15817207.

31. Panic G, Flores D, Ingram-Sieber K, Keiser J. Fluorescence/luminescence-based markers for the assessment of *Schistosoma mansoni* schistosomula drug assays. Parasit Vectors. 2015;8:624. Epub 2015/12/09. doi: 10.1186/s13071-015-1233-3

10.1186/s13071-015-1233-3 [pii]. PubMed PMID: 26644133; PubMed Central PMCID: PMC4672532.

32. Peak E, Chalmers IW, Hoffmann KF. Development and validation of a quantitative, high-throughput, fluorescent-based bioassay to detect schistosoma viability. PLoS Negl Trop Dis. 2010;4(7):e759. Epub

2010/07/30. doi: 10.1371/journal.pntd.0000759. PubMed PMID: 20668553; PubMed Central PMCID: PMC2910722.

33. Almeida GT, Lage RC, Anderson L, Venancio TM, Nakaya HI, Miyasato PA, et al. Synergy of Omeprazole and Praziquantel In Vitro Treatment against *Schistosoma mansoni* Adult Worms. PLoS Negl Trop Dis. 2015;9(9):e0004086. Epub 2015/09/25. doi: 10.1371/journal.pntd.0004086

PNTD-D-15-00436 [pii]. PubMed PMID: 26402251; PubMed Central PMCID: PMC4581627.

34. Storey DM, Ogbogu VC. Observations on third-stage larvae and adults of Litomosoides carinii (Nematoda: Filarioidea) by scanning and transmission electron microscopy. Ann Trop Med Parasitol. 1991;85(1):111-21. Epub 1991/02/01. PubMed PMID: 1888208.

35. Cogswell AA, Kommer VP, Williams DL. Transcriptional analysis of a unique set of genes involved in *Schistosoma mansoni* female reproductive biology. PLoS Negl Trop Dis. 2012;6(11):e1907. Epub 2012/11/21. doi: 10.1371/journal.pntd.0001907

PNTD-D-12-00991 [pii]. PubMed PMID: 23166854; PubMed Central PMCID: PMC3499410.

36. Pereira RV, Gomes MdS, Olmo RP, Souza DM, Cabral FJ, Jannotti-Passos LK, et al. Ubiquitin-specific proteases are differentially expressed throughout the *Schistosoma mansoni* life cycle. Parasit Vectors. 2015;8:349. Epub 2015/06/27. doi: 10.1186/s13071-015-0957-4

10.1186/s13071-015-0957-4 [pii]. PubMed PMID: 26112833; PubMed Central PMCID: PMC4485857.

37. Farias LP, Tararam CA, Miyasato PA, Nishiyama MY, Jr., Oliveira KC, Kawano T, et al. Screening the *Schistosoma mansoni* transcriptome for genes differentially expressed in the schistosomulum stage in search for vaccine candidates. Parasitol Res. 2011;108(1):123-35. Epub 2010/09/21. doi: 10.1007/s00436-010-2045-1. PubMed PMID: 20852890.

38. Pereira ASA, Amaral MS, Vasconcelos EJR, Pires DS, Asif H, daSilva LF, et al. Inhibition of histone methyltransferase EZH2 in *Schistosoma mansoni* in vitro by GSK343 reduces egg laying and decreases the expression of genes implicated in DNA replication and noncoding RNA metabolism. PLoS Negl Trop Dis. 2018;12(10):e0006873. Epub 2018/10/27. doi: 10.1371/journal.pntd.0006873

PNTD-D-18-01081 [pii]. PubMed PMID: 30365505; PubMed Central PMCID: PMC6221359.

39. Anderson L, Gomes MR, daSilva LF, Pereira A, Mourao MM, Romier C, et al. Histone deacetylase inhibition modulates histone acetylation at gene promoter regions and affects genome-wide gene transcription in *Schistosoma mansoni*. PLoS Negl Trop Dis. 2017;11(4):e0005539. Epub 2017/04/14. doi: 10.1371/journal.pntd.0005539

PNTD-D-16-02315 [pii]. PubMed PMID: 28406899; PubMed Central PMCID: PMC5404884.

40. Marek M, Kannan S, Hauser AT, Moraes Mourao M, Caby S, Cura V, et al. Structural basis for the inhibition of histone deacetylase 8 (HDAC8), a key epigenetic player in the blood fluke *Schistosoma mansoni*. PLoS Pathog. 2013;9(9):e1003645. Epub 2013/10/03. doi: 10.1371/journal.ppat.1003645

PPATHOGENS-D-13-01384 [pii]. PubMed PMID: 24086136; PubMed Central PMCID: PMC3784479.

41. Wang J, Collins JJ, 3rd. Identification of new markers for the *Schistosoma mansoni* vitelline lineage. International journal for parasitology. 2016;46(7):405-10. Epub 2016/04/09. doi: 10.1016/j.ijpara.2016.03.004. PubMed PMID: 27056273; PubMed Central PMCID: PMC4917872.

42. Utzinger J, Raso G, Brooker S, De Savigny D, Tanner M, Ornbjerg N, et al. Schistosomiasis and neglected tropical diseases: towards integrated and sustainable control and a word of caution.

Parasitology. 2009;136(13):1859-74. Epub 2009/11/13. doi: 10.1017/S0031182009991600. PubMed PMID: 19906318; PubMed Central PMCID: PMCPMC2791839.

43. Carvalho O, Coelho P, Lenzi L. Terapêutica experimental da esquistossomose mansoni. *Schistosoma mansoni* e esquistossomose: uma visão multidisciplinar Rio de Janeiro: Editora Fiocruz; 2008. p. 822–47.

44. de Oliveira RN, Rehder VL, Santos Oliveira AS, Junior IM, de Carvalho JE, de Ruiz AL, et al. *Schistosoma mansoni*: in vitro schistosomicidal activity of essential oil of Baccharis trimera (less) DC. Exp Parasitol. 2012;132(2):135-43. Epub 2012/07/10. doi: 10.1016/j.exppara.2012.06.005. PubMed PMID: 22771865.

45. Moraes J, Nascimento C, Lopes PO, Nakano E, Yamaguchi LF, Kato MJ, et al. *Schistosoma mansoni:* In vitro schistosomicidal activity of piplartine. Exp Parasitol. 2011;127(2):357-64. Epub 2010/09/14. doi: 10.1016/j.exppara.2010.08.021. PubMed PMID: 20832410.

46. Kumar R, Mohanakrishnan D, Sharma A, Kaushik NK, Kalia K, Sinha AK, et al. Reinvestigation of structure-activity relationship of methoxylated chalcones as antimalarials: synthesis and evaluation of 2,4,5-trimethoxy substituted patterns as lead candidates derived from abundantly available natural beta-asarone. Eur J Med Chem. 2010;45(11):5292-301. Epub 2010/09/25. doi: 10.1016/j.ejmech.2010.08.049. PubMed PMID: 20863599.

47. Bello ML, Chiaradia LD, Dias LR, Pacheco LK, Stumpf TR, Mascarello A, et al. Trimethoxy-chalcone derivatives inhibit growth of *Leishmania braziliensis*: synthesis, biological evaluation, molecular modeling and structure-activity relationship (SAR). Bioorg Med Chem. 2011;19(16):5046-52. Epub 2011/07/16. doi: 10.1016/j.bmc.2011.06.023. PubMed PMID: 21757358.

48. Bernardes LS, Kato MJ, Albuquerque S, Carvalho I. Synthesis and trypanocidal activity of 1,4-bis-(3,4,5-trimethoxy-phenyl)-1,4-butanediol and 1,4-bis-(3,4-dimethoxyphenyl)-1,4-butanediol. Bioorg Med Chem. 2006;14(21):7075-82. Epub 2006/08/16. doi: 10.1016/j.bmc.2006.07.006. PubMed PMID: 16908164.

49. Papadopoulou MV, Bloomer WD, Rosenzweig HS, Wilkinson SR, Szular J, Kaiser M. Antitrypanosomal activity of 5-nitro-2-aminothiazole-based compounds. Eur J Med Chem. 2016;117:179-86. Epub 2016/04/20. doi: 10.1016/j.ejmech.2016.04.010. PubMed PMID: 27092415; PubMed Central PMCID: PMCPMC4876673.

50. Paula FR, Serrano SHP, Tavares LC. Aspects of Bioactivity and Toxicity of Nitrocompounds. Quim Nova. 2009;32(4):1013-20. doi: Doi 10.1590/S0100-40422009000400032. PubMed PMID: WOS:000267331400032.

51. Rai G, Thomas CJ, Leister W, Maloney DJ. Synthesis of Oxadiazole-2-oxide Analogues as Potential Antischistosomal Agents. Tetrahedron Lett. 2009;50(15):1710-3. Epub 2009/10/06. PubMed PMID: 19802367; PubMed Central PMCID: PMCPMC2721338.

52. Gillis EP, Eastman KJ, Hill MD, Donnelly DJ, Meanwell NA. Applications of Fluorine in Medicinal Chemistry. J Med Chem. 2015;58(21):8315-59. Epub 2015/07/23. doi: 10.1021/acs.jmedchem.5b00258. PubMed PMID: 26200936.

53. Altintop MD, Atli O, Ilgin S, Demirel R, Ozdemir A, Kaplancikli ZA. Synthesis and biological evaluation of new naphthalene substituted thiosemicarbazone derivatives as potent antifungal and anticancer agents. Eur J Med Chem. 2016;108:406-14. Epub 2015/12/27. doi: 10.1016/j.ejmech.2015.11.041. PubMed PMID: 26706351.

54. Greenberg RM. Are Ca2+ channels targets of praziquantel action? International journal for parasitology. 2005;35(1):1-9. Epub 2004/12/28. doi: 10.1016/j.ijpara.2004.09.004. PubMed PMID: 15619510.

55. Harder A, Goossens J, Andrews P. Influence of praziquantel and Ca2+ on the bilayer-isotropic-hexagonal transition of model membranes. Mol Biochem Parasitol. 1988;29(1):55-9. Epub 1988/05/01. PubMed PMID: 3386687.

56. Lima SF, Vieira LQ, Harder A, Kusel JR. Effects of culture and praziquantel on membrane fluidity parameters of adult *Schistosoma mansoni*. Parasitology. 1994;109 (Pt 1):57-64. Epub 1994/07/01. PubMed PMID: 8058369.

57. Orrenius S, Zhivotovsky B, Nicotera P. Regulation of cell death: The calcium-apoptosis link. Nat Rev Mol Cell Bio. 2003;4(7):552-65. doi: 10.1038/nrm1150. PubMed PMID: WOS:000183892000014.

58. Pinton P, Giorgi C, Siviero R, Zecchini E, Rizzuto R. Calcium and apoptosis: ER-mitochondria Ca2+ transfer in the control of apoptosis. Oncogene. 2008;27(50):6407-18. Epub 2008/10/29. doi: 10.1038/onc.2008.308. PubMed PMID: 18955969; PubMed Central PMCID: PMCPMC2844952.

59. You H, McManus DP, Hu W, Smout MJ, Brindley PJ, Gobert GN. Transcriptional responses of in vivo praziquantel exposure in schistosomes identifies a functional role for calcium signalling pathway member CamKII. PLoS Pathog. 2013;9(3):e1003254. Epub 2013/04/05. doi: 10.1371/journal.ppat.1003254. PubMed PMID: 23555262; PubMed Central PMCID: PMC3610926.

60. Marxer M, Ingram K, Keiser J. Development of an in vitro drug screening assay using *Schistosoma haematobium* schistosomula. Parasit Vectors. 2012;5:165. Epub 2012/08/11. doi: 10.1186/1756-3305-5-165. PubMed PMID: 22876861; PubMed Central PMCID: PMCPMC3437206.

61. Bertao HG, da Silva RAR, Padilha RJR, Albuquerque MCPA, Radis-Baptista G. Ultrastructural analysis of miltefosine-induced surface membrane damage in adult *Schistosoma mansoni* BH strain worms. Parasitology Research. 2012;110(6):2465-73. doi: 10.1007/s00436-011-2786-5. PubMed PMID: WOS:000304627300046.

62. Taha H, Soliman M. Antischistosomal Activity of 3-Substituted-5-(2-Aryl-2-Oxoethyl) -2, 4-Dioxo-1, 3-Thiazolidine (Ro-354) INTERNATIONAL JOURNAL OF AGRICULTURE &BIOLOGY. 2007;09(1):87–93.

63. Mohamed S, Fawzi S. Scanning electron microscopy on adults of *Schistosoma mansoni* treated in vivo with praziquantel and Ro-15(5458). . Qatar University Sci J 1997;17:439–58.

64. Fawzi S. Ultrastructural studies on the effect of antischistosomal drug Ro15-5458 on the tegument of male *Schistosoma mansoni*. Egypt Zool 1999;33:21–31.

65. Manneck T, Haggenmuller Y, Keiser J. Morphological effects and tegumental alterations induced by mefloquine on schistosomula and adult flukes of *Schistosoma mansoni*. Parasitology. 2010;137(1):85-98. Epub 2009/10/10. doi: 10.1017/S0031182009990965. PubMed PMID: 19814844.

66. Xiao S, Shen B, Utzinger J, Chollet J, Tanner M. Ultrastructural alterations in adult *Schistosoma mansoni* caused by artemether. Mem Inst Oswaldo Cruz. 2002;97(5):717-24. Epub 2002/09/10. PubMed PMID: 12219141.

67. Fitzpatrick JM, Hirai Y, Hirai H, Hoffmann KF. Schistosome egg production is dependent upon the activities of two developmentally regulated tyrosinases. FASEB J. 2007;21(3):823-35. Epub 2006/12/15. doi: 10.1096/fj.06-7314com. PubMed PMID: 17167065.

68. Bobek LA, Rekosh DM, LoVerde PT. Small gene family encoding an eggshell (chorion) protein of the human parasite *Schistosoma mansoni*. Mol Cell Biol. 1988;8(8):3008-16. Epub 1988/08/01. PubMed PMID: 2850476; PubMed Central PMCID: PMCPMC363526.

69. Chen LL, Rekosh DM, LoVerde PT. *Schistosoma mansoni* p48 eggshell protein gene: characterization, developmentally regulated expression and comparison to the p14 eggshell protein gene. Mol Biochem Parasitol. 1992;52(1):39-52. Epub 1992/05/01. PubMed PMID: 1625706.

70. Nollen PM, Floyd RD, Kolzow RG, Deter DL. The timing of reproductive cell development and movement in *Schistosoma mansoni*, *S. japonicum*, and *S. haematobium*, using techniques of autoradiography and transplantation. J Parasitol. 1976;62(2):227-31. Epub 1976/04/01. PubMed PMID: 1263031.

71. Seydoux G, Braun RE. Pathway to totipotency: lessons from germ cells. Cell. 2006;127(5):891-904. Epub 2006/11/30. doi: 10.1016/j.cell.2006.11.016. PubMed PMID: 17129777.

72. Wang Y, Zayas RM, Guo T, Newmark PA. nanos function is essential for development and regeneration of planarian germ cells. Proc Natl Acad Sci U S A. 2007;104(14):5901-6. Epub 2007/03/23. doi: 10.1073/pnas.0609708104. PubMed PMID: 17376870; PubMed Central PMCID: PMCPMC1851589.

73. Wang YY, Stary JM, Wilhelm JE, Newmark PA. A functional genomic screen in planarians identifies novel regulators of germ cell development. Gene Dev. 2010;24(18):2081-92. doi: 10.1101/gad.1951010. PubMed PMID: WOS:000281815500010.

74. Ribeiro-dos-Santos G, Verjovski-Almeida S, Leite LC. Schistosomiasis--a century searching for chemotherapeutic drugs. Parasitol Res. 2006;99(5):505-21. Epub 2006/04/26. doi: 10.1007/s00436-006-0175-2. PubMed PMID: 16636847.

Supporting Information Legends

Fig A. ATP quantitation using a luminescent assay to assess schistosomula survival under NJ series compounds exposure. Schistosomula (100-120/well) were incubated with the indicated concentrations of NJ03, NJ04, NJ06, NJ08, NJ11 or NJ12 or with control vehicle (0.1% DMSO) for up to 5 days. Viability is expressed as % luminescence values relative to the control (DMSO). Mean ± SEM from three replicate experiments. *p < 0.05 (two-way ANOVA). For clarity purposes, we show only the highest p-value obtained from the two-way ANOVA test for each time point on all different concentrations tested.

Fig B. Effect of NJ series compounds on the viability of *Schistosoma mansoni* adult worms. Viability was estimated by the total amount of ATP available in the parasites, using a luminescent assay. Pairs of adult worms were treated for 1 to 3 days with NJ03, NJ04, NJ06, NJ08, NJ12 and NJ12 at the different concentrations indicated or with vehicle (0.1% DMSO). Viability was expressed as % luminescence values relative to the control (0.1% DMSO). Mean ± SEM from three replicate experiments, each with 10 worm pairs. *p < 0.05 compared with control. For clarity purposes, we show only the highest p-value obtained from the two-way ANOVA test for each time point on all different concentrations tested

Fig C. Effect of NJ series compounds on the motility of *Schistosoma mansoni* adult worms. Percentage of relative motility of adult worms treated with different concentrations of NJ03, NJ04, NJ06, NJ08, NJ12 and NJ12 and controls (0.1 % DMSO) at different times of exposure from 1 to 3 days. Mean ± SEM of three experiments, each with 10 worm pairs. *p < 0.05, **p < 0.01 compared with control. For clarity purposes, we show only the highest p-value obtained from the two-way ANOVA test for each time point on all different concentrations tested.

Fig D. *In vitro* effect of NJ series compounds on pairing of *Schistosoma mansoni* adult worm couples. Monitoring the pairing status of control couples and of treated couples exposed to different concentrations of NJ03, NJ04, NJ06, NJ08, NJ11 and NJ12 at different times of exposure from 1 to 3 days. Mean ± SEM of three experiments, each with 10 worm pairs. *p < 0.05 and **p <

0.01 compared with control. For clarity purposes, we show only the highest p-value obtained from the two-way ANOVA test for each time point on all different concentrations tested.

Fig E. *In vitro* effects of NJ series compounds on oviposition of *Schistosoma mansoni* adult worms. Number of eggs released by females incubated with NJ03, NJ04, NJ06, NJ08, NJ11 and NJ12 at different concentrations or with vehicle (0.1 % DMSO) at different times of exposure from 1 to 3 days. Mean ± SEM of three experiments, each with 10 worm pairs. $^*p < 0.05$ and $^{**}p < 0.01$ compared with control. For clarity purposes, we show only the highest p-value obtained from the two-way ANOVA test for each time point on all different concentrations tested.

Fig F. Structural damage was observed by scanning electron microscopy of male worms already at 24 h exposure to NJ05 or NJ07. Worms were exposed for 24 h to 25 μM NJ05, 25 μM NJ07 or to vehicle (0.1% DMSO). **panels 1-4**: Control worms (Bar = 500 μm; 100 μm; 50 μm; 50 μm) presenting the fully paired couple (panel 2) and anterior region of the male presenting normal oral and ventral suckers tegument (panels 3 and 4); **panels 5-8**: Anterior region of adult male worm treated with NJ05 with oral and ventral suckers presenting tegument peeling (Bar = 500 μm; 100 μm; 50 μm; 50 μm); **panels 9-12**: Anterior region of adult male worm treated with NJ07 with oral and ventral suckers presenting tegument without structural alterations (Bar = 400 μm; 100 μm; 50 μm; 40 μm); **panels 13-16**: Medial (panels 13 – 15) and posterior (panel 16) regions of control male worm presenting normal tegument structures (Bar = 50 μm; 20 μm; 5 μm; 50 μm); **panels 17-20:** Enlarged view of dorsal region of adult worm treated with NJ05 (panels 17-19) showing tegument lesions and loss of tubercles and posterior region of male adult worm showing lesion areas (Bar = 50 μm; 20 μm; 5 μm; 50 μm); **panels 21-24:** Dorsal and posterior regions of adult worm treated with NJ07 showing bubbles along their extension (Bar = 30 μm; 20 μm; 5 μm; 50 μm). *f*: *female; os*: *oral sucker; vs*: *ventral sucker; gc: gynecophoral canal*; *la*: *lesion area* ; *cp: ciliated papillae; b: blebs; tb*: *tubercles*. (note that the size scale bar is shown within the black thin line below each image).

Fig G: Vitellaria and female reproduction gene expression in adult females upon NJ07 or NJ05+NJ07 treatment. Adult couples were treated with NJ07 (25 μM), NJ05+NJ07 (25 μM) or DMSO (0.1 %) for 2 days. The parasites were stored in RNAlater for further processing. All parasite couples were separated and only the females had their RNA extracted followed by cDNA synthesis. The genes measured by qPCR were Smp_131110 (p14), Smp_000430 (Egg Shell Protein (ESP)), Smp_013540 (Tyrosinase 2 (Tyr 2)), Smp_014610 (p48), Smp_000290 (fs800), Smp_055740 (Nanos 2). The geometric mean from two reference genes was used for normalization with the DCT method (Smp_090920 and Smp_123610). The plotted data is retrieved from DDCT analyses in which DMSO sample is the control. Significant fold-change in gene expression between DMSO and NJ07 or NJ05+NJ07 treatment is shown by the numbers inside the brackets. Student unpaired parametric two-sided t-test was used and statistically significant differences are represented by the asterisks. *$p \leq 0.05$; **$p \leq 0.01$.

S1 Table. List of primers used in qPCR

Figure 1

Click here to access/download;Figure;Figure 1.tif

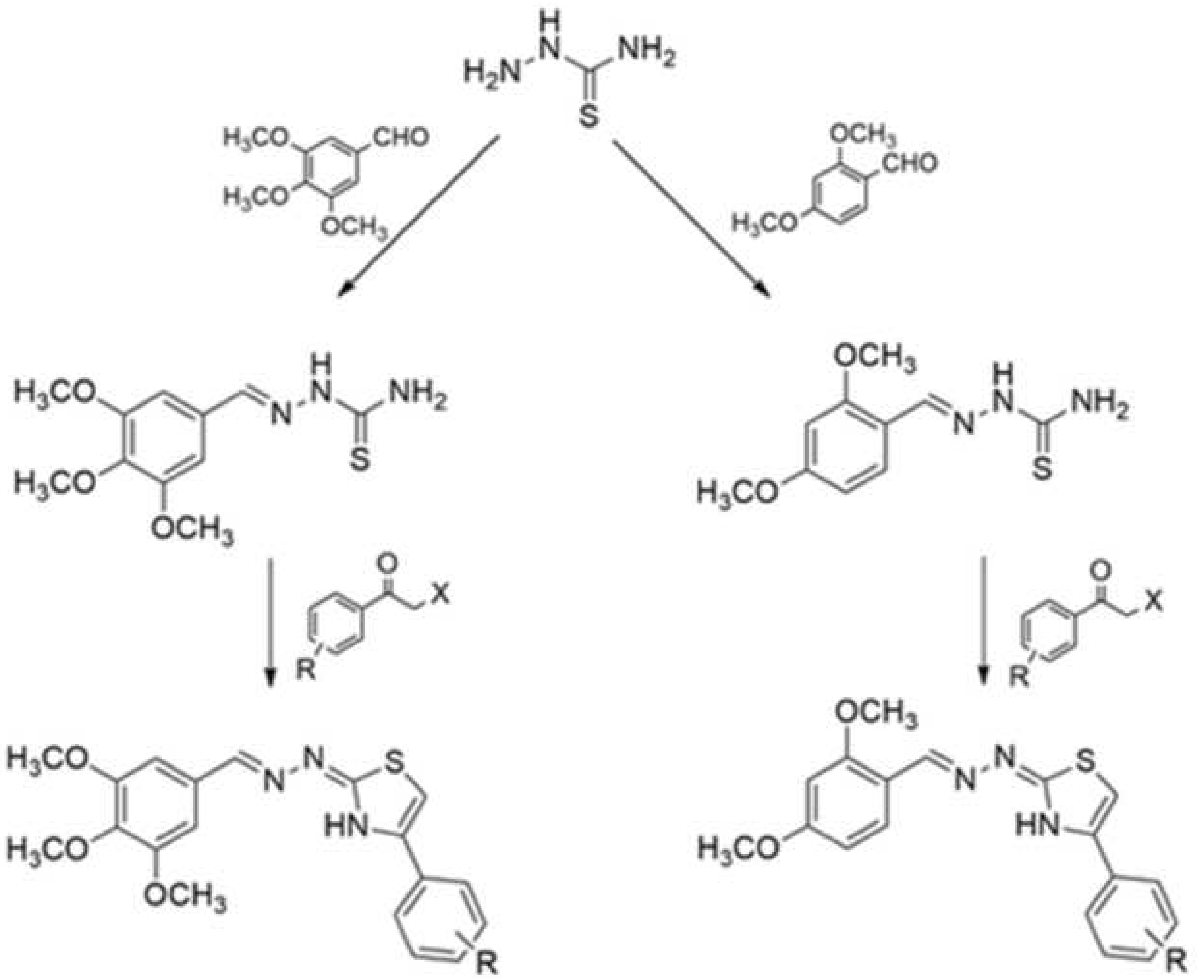

Figure 2

Click here to access/download;Figure;Figure 2.tif

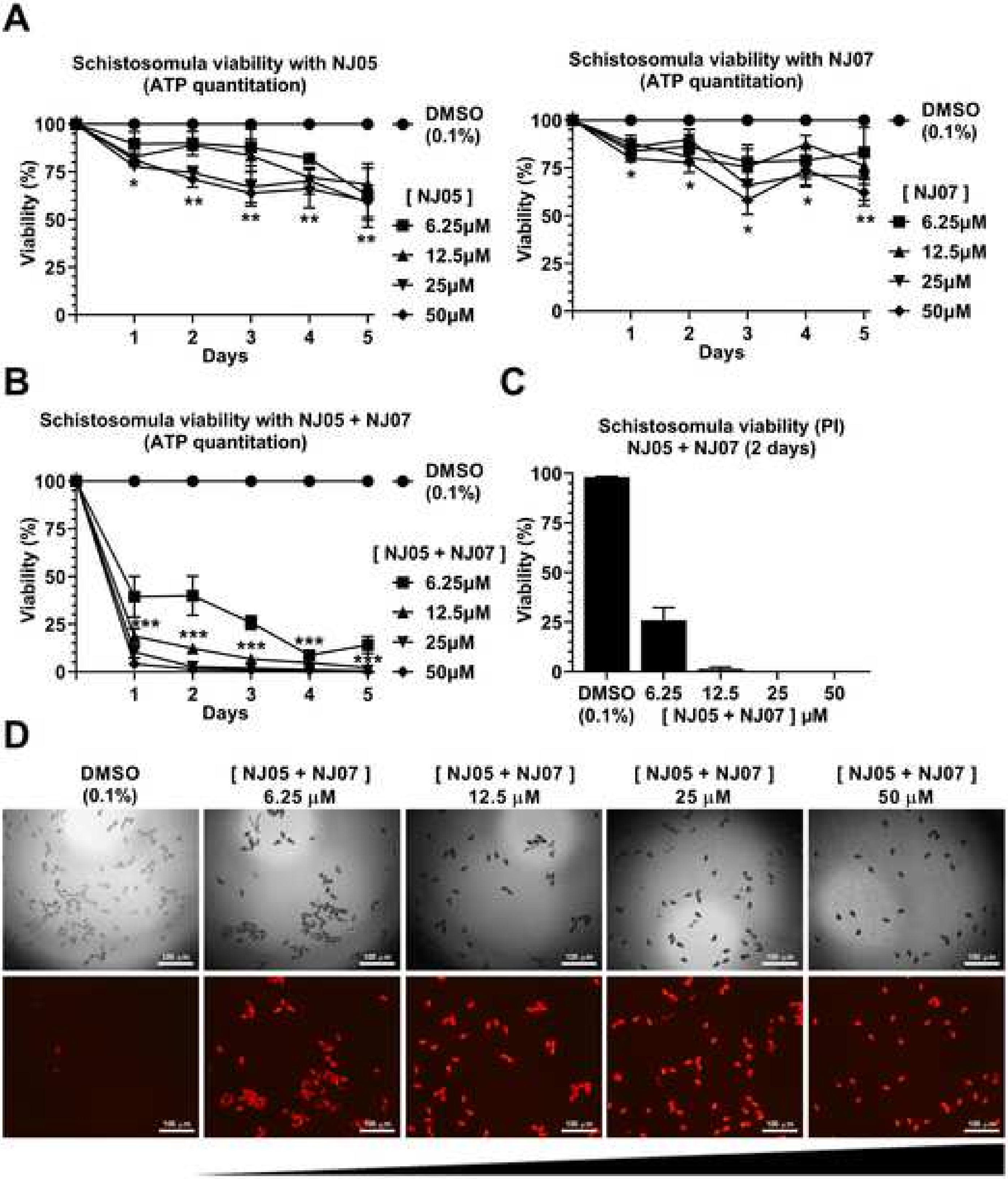

Figure 3

Click here to access/download;Figure;Figure 3.tif

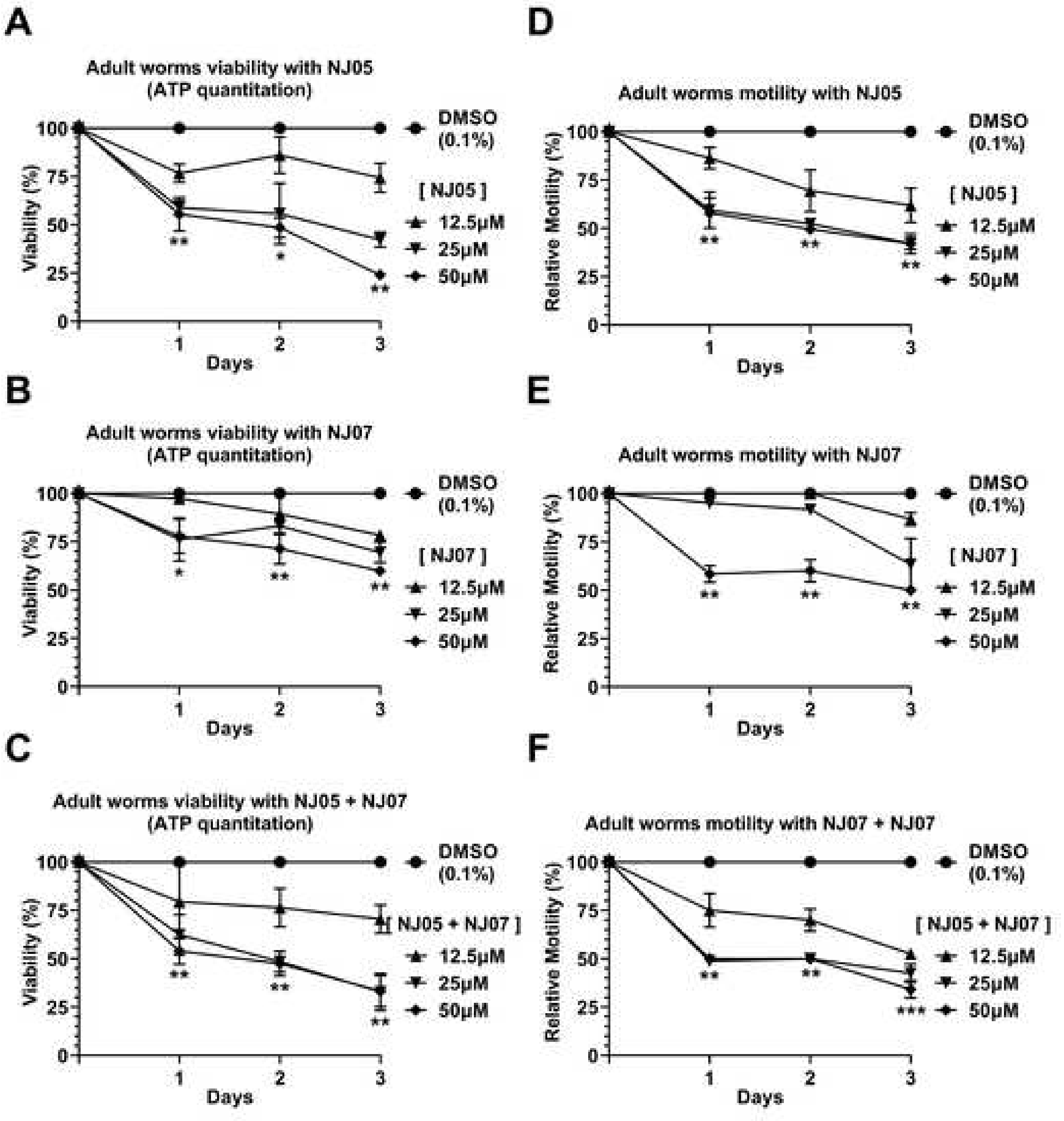

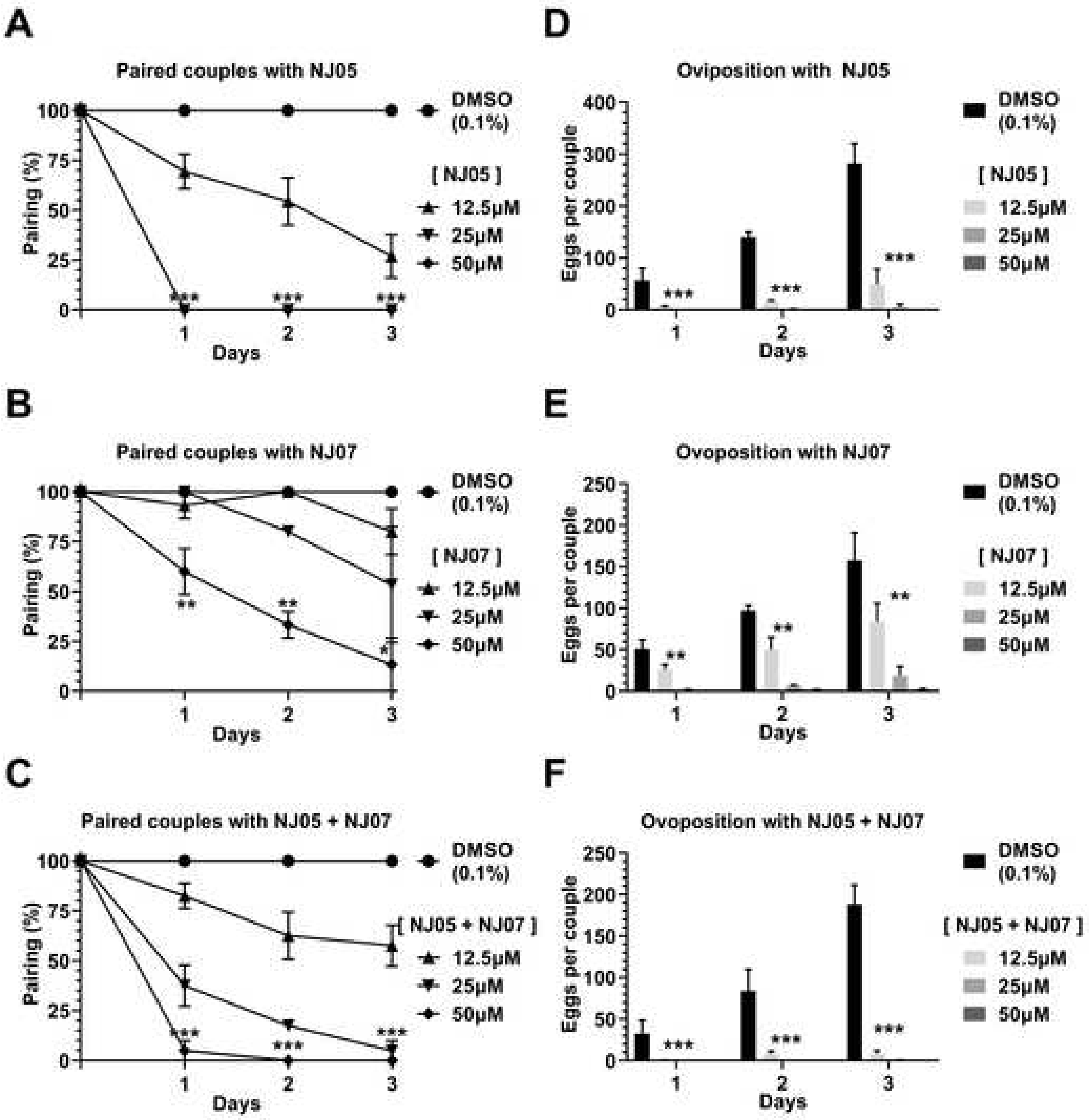
A
Paired couples with NJ05
Pairing (%)
Days
DMSO (0.1%)
[NJ05]
12.5μM
25μM
50μM
***
D
Oviposition with NJ05
Eggs per couple
Days
B
Paired couples with NJ07
[NJ07]
**
*
E
Ovoposition with NJ07
C
Paired couples with NJ05 + NJ07
[NJ05 + NJ07]
F
Ovoposition with NJ05 + NJ07

Figure 5

Click here to access/download;Figure;Figure 5.tif

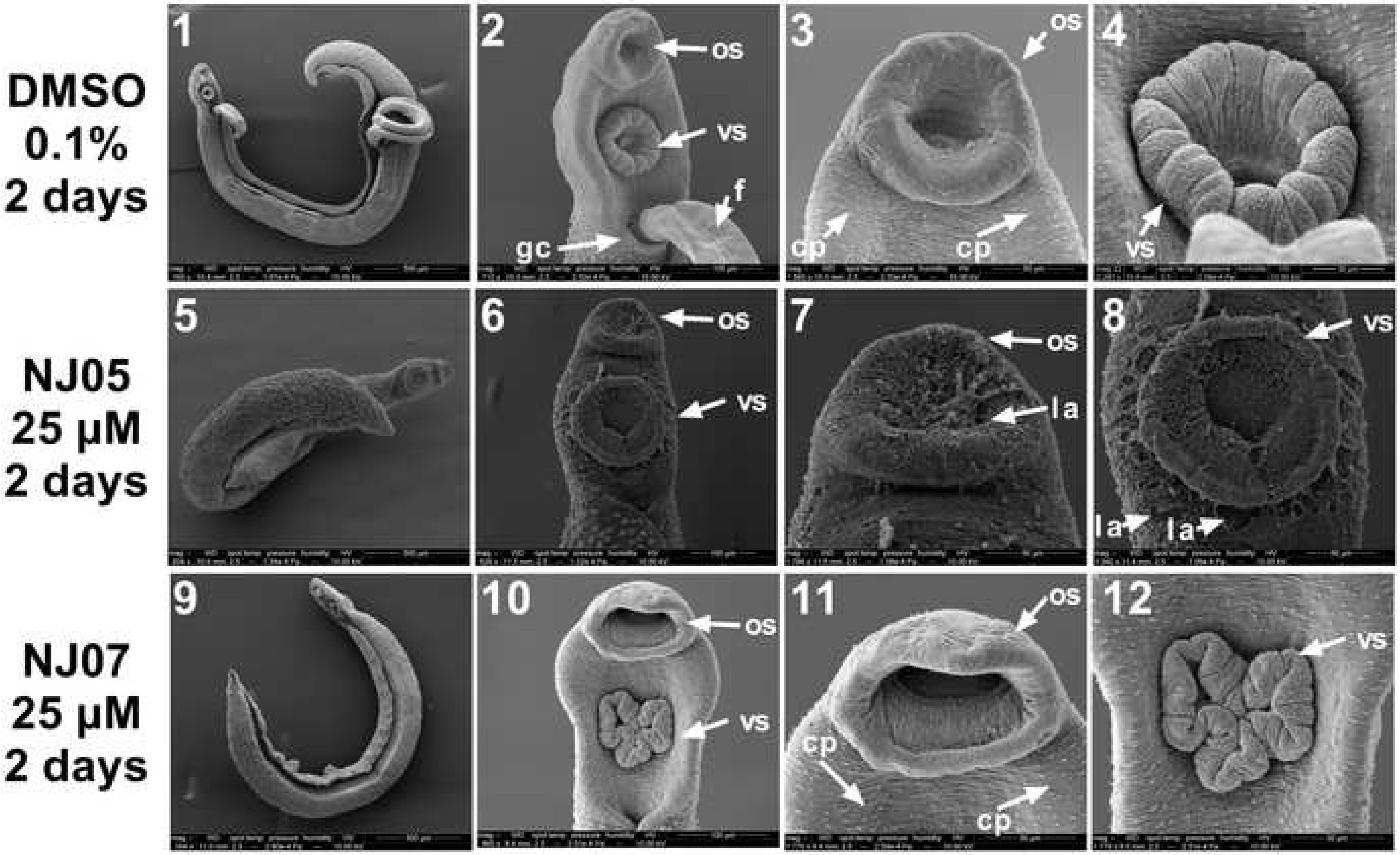

Figure 6

Click here to access/download;Figure;Figure 6.tif

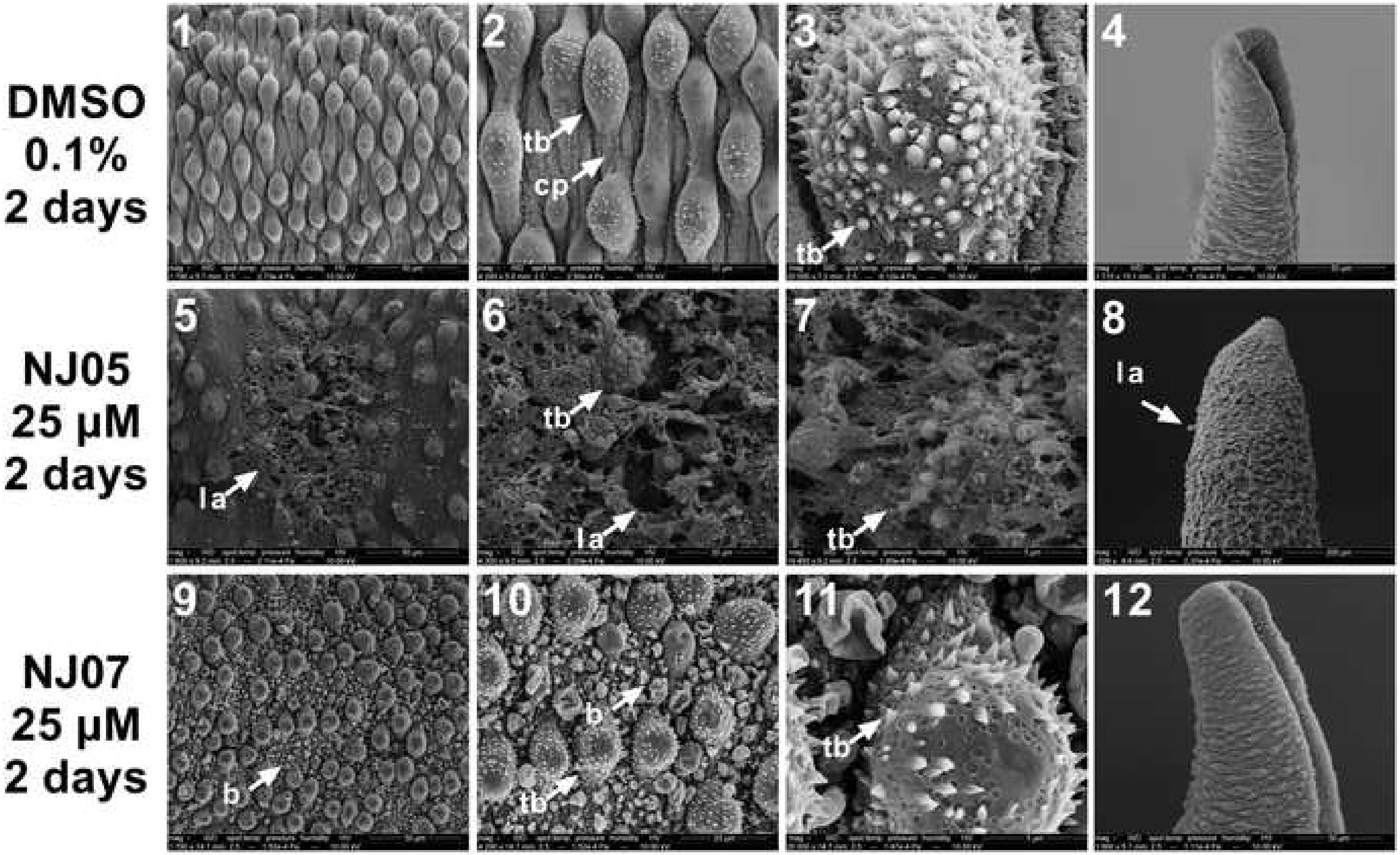

Click here to access/download;Figure;Figure 7.tif

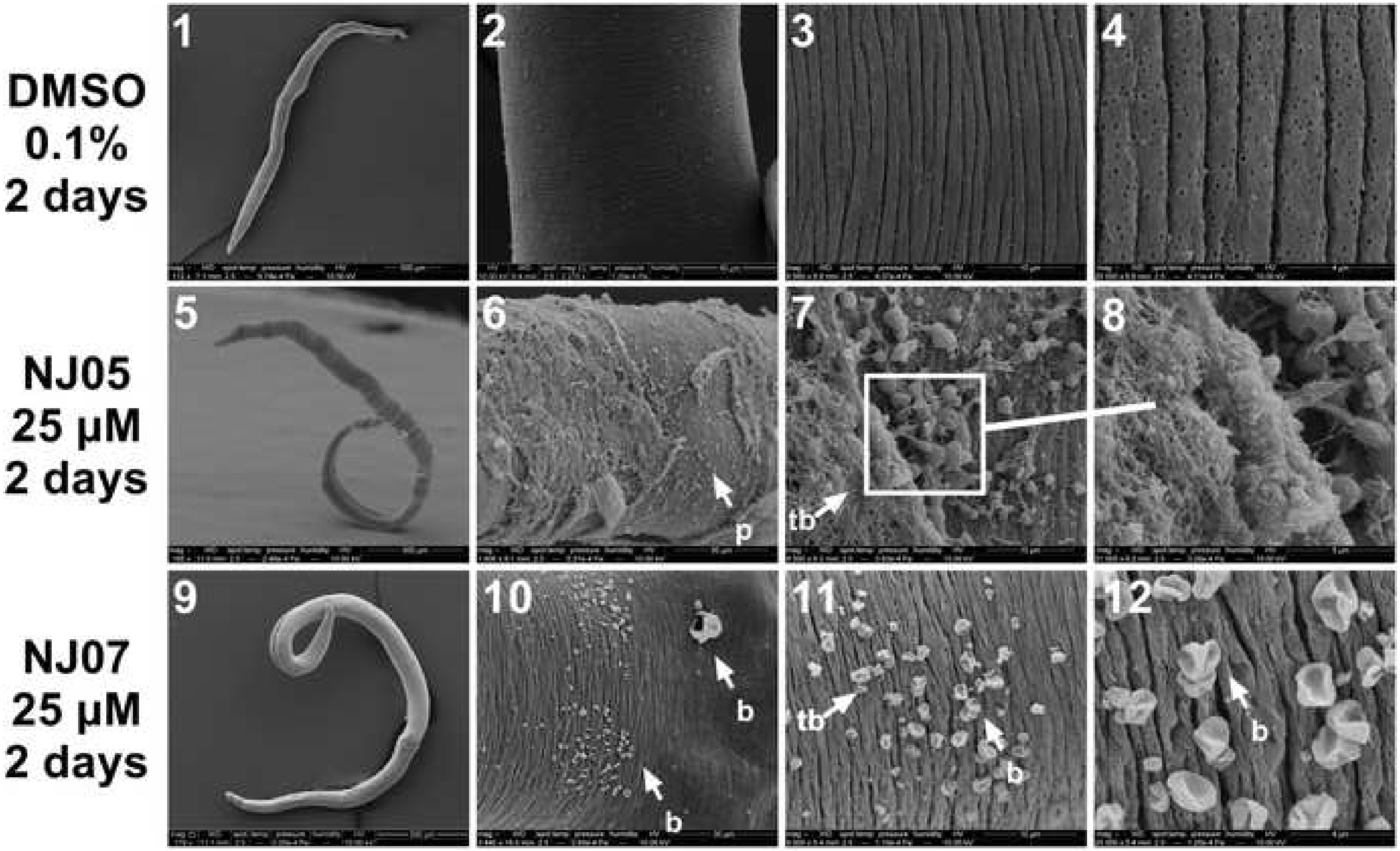

Figure 8

Click here to access/download;Figure;Figure 8.tif

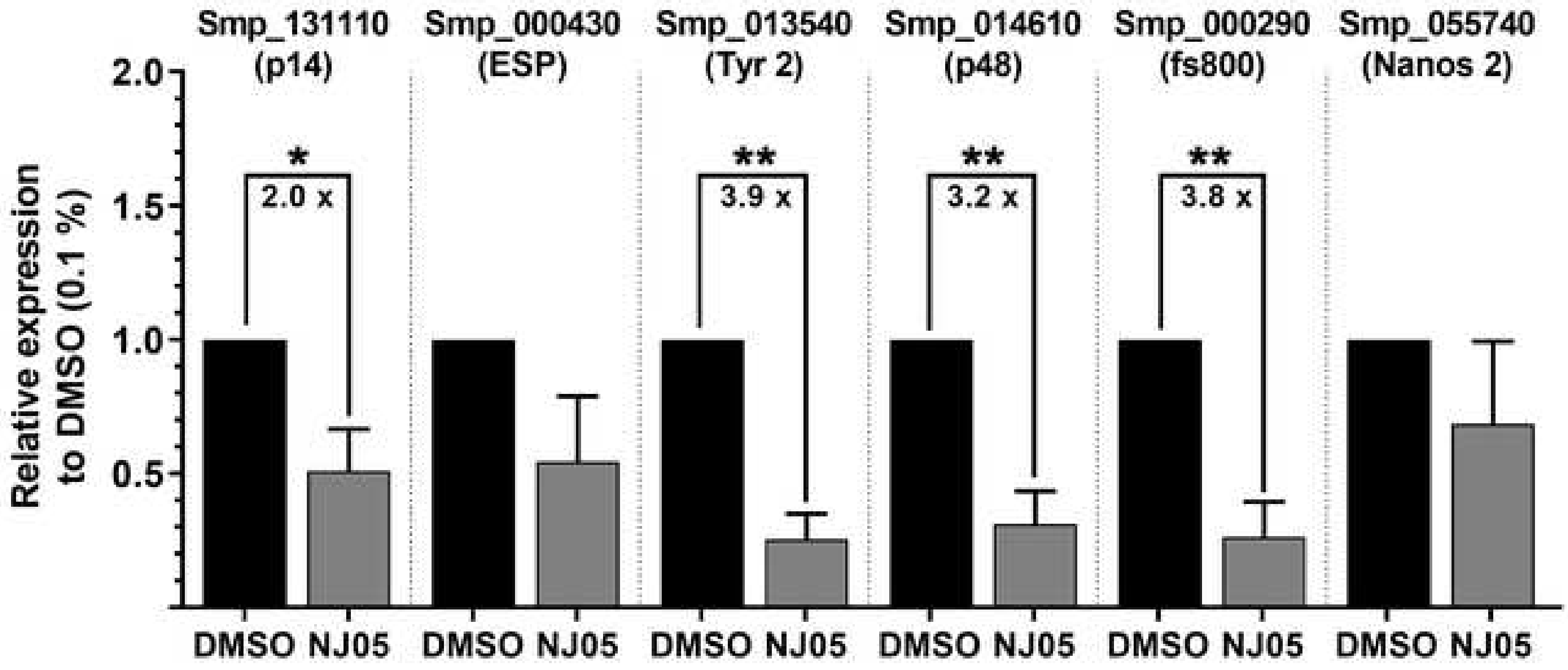

S1 Table - List of primers used in qPCR

Click here to access/download
Supporting Information
S1_Table. List of primers used in qPCR.xlsx

S1 text Supporting information

Click here to access/download
Supporting Information
S1_Text.docx
