# Supplementary material for: In vitro activity of aryl-thiazole derivatives against Schistosoma mansoni schistosomula and adult worms
Source: PLoS One. 2019 Nov 25;14(11):e0225425. doi: 10.1371/journal.pone.0225425 (PMC6876889; doi:10.1371/journal.pone.0225425)
Supplement: Attachment — Submitted filename: Pereira et al review Vermeire.docx [file pone.0225425.s004.docx]

Pereira *et al.* *In vitro* activity of aryl-thiazole derivatives against *Schistosoma mansoni* schistosomula and adult worms PLOS One review

In this manuscript the authors report their work on synthesizing a series of thiazole derivatives and the assessment of the anthelmintic activity of 8 of these compounds *in* *vitro*. I feel that this description of their work is very thorough and their study design draws upon all of the best molecular techniques available to synthesize, interrogate and execute these particular class of studies. Their assessments of parasite motility and mortality, egg laying, adult worm pairing and parasite viability clearly demonstrate the antischistosomal activity of their compounds, especially compounds NJ05 and NJ07. The antischistosomal activity of compound NJ05 against juvenile worms shows particular promise given the lack of activity by the gold standard of treatment, praziquantel. The only reason for my suggestion of minor revision include some grammatical issues in the text (e.g. verb tense).
