# Supplementary material for: In vitro activity of aryl-thiazole derivatives against Schistosoma mansoni schistosomula and adult worms
Source: PLoS One. 2019 Nov 25;14(11):e0225425. doi: 10.1371/journal.pone.0225425 (PMC6876889; doi:10.1371/journal.pone.0225425)
Supplement: Attachment — Submitted filename: Point-by-point responses to Referees.docx [file pone.0225425.s005.docx]

**Point-by-point responses to the Referees’ comments:**

**Reviewer #1:** This manuscript described the schistosomicidal activities of aryl-thiazole derivatives. Some of the data and expression in this manuscript are unclear and should be revised before it can be accepted for publication. My comments are listed below:

1- *The manuscript is very difficult to read. Please organize the Material and Methods session. e.g. Title: "Treatment of adult worms with NJ series compounds".*

*Paragraph: "Schistosomula and adult worms were treated with different concentrations of compounds from the NJ series in culture medium specific to each stage as indicated below..." (page 13). According to the results, adult worms and schistosomula treatments were performed under different conditions (incubation period), please make this information clear in the text;*

R- Following the Referee’s request, we have corrected all Titles in the Methods section in which there was only partial information. In addition, we have modified the text in the section “Treatment of schistosomula and adult worms with NJ series compounds” (pages 9-10) in order to add the missing information about concentration and different assay conditions for schistosomula versus adults.

2- *It's very important to remind the necessity of cytotoxicity assay done to prove the absence of toxicity of the aryl-thiazole derivatives, before this manuscript to be accepted for publication;*

R- As requested by the Referee, we have now added on page 17 the results of cytotoxicity evaluation of NJ05, NJ07 and NJ05 + NJ07 on HEK293 cell line (Human Embryo Kidney- HEK-293 -ATCC® CRL-1573™) and HES cell line (endometrial epithelial cell line, kindly provided by Prof. Douglas Anthony Kniss - Ohio State University, USA).

3- *It’s widely studied the correlation between the effect of some drugs on S. mansoni motility through of classical studies. e.g. Hillman, G. R., Senft, A.W.J Pharmacol Exp Ther (1993), 119, 75-86.; Da Silva, S.P., Noel, E. Parasitol Res (1995), 81:543-548; Mendonça Silva, D. L. et al., Parasitology (2004), 129 : 1-10; Rinaldi, G. et al., Int J Parasitol-Drug (2015), 141e148; De Oliveira, R.N., Exp Parasitol (2012), 132,135–143.
In present study, motility and survival of worms were assessed according to the criteria of a nematode (Toxocara canis- ref 30), which are completely different from trematode. Please, why this choice? (Page 14, session “Motility assay");*

R- We have used the Horiuchi et al. method (*Toxocara canis* – now ref 29) because it is a method optimized to quantitatively assess larvae motility based on their movement within a time window. This scoring method has been adapted and is also used to evaluate the mobility of trematode parasites as described in the literature: Aires Ade L, [Ximenes EC](https://www.ncbi.nlm.nih.gov/pubmed/?term=Ximenes%20EC%5BAuthor%5D&cauthor=true&cauthor_uid=24768955), [Silva RA](https://www.ncbi.nlm.nih.gov/pubmed/?term=Silva%20RA%5BAuthor%5D&cauthor=true&cauthor_uid=24768955), [Barbosa VX](https://www.ncbi.nlm.nih.gov/pubmed/?term=Barbosa%20VX%5BAuthor%5D&cauthor=true&cauthor_uid=24768955), [Góes AJ](https://www.ncbi.nlm.nih.gov/pubmed/?term=G%C3%B3es%20AJ%5BAuthor%5D&cauthor=true&cauthor_uid=24768955), [Peixoto CA](https://www.ncbi.nlm.nih.gov/pubmed/?term=Peixoto%20CA%5BAuthor%5D&cauthor=true&cauthor_uid=24768955), [Souza VM](https://www.ncbi.nlm.nih.gov/pubmed/?term=Souza%20VM%5BAuthor%5D&cauthor=true&cauthor_uid=24768955), [Albuquerque MC](https://www.ncbi.nlm.nih.gov/pubmed/?term=Albuquerque%20MC%5BAuthor%5D&cauthor=true&cauthor_uid=24768955). Ultrastructural analysis of β-lapachone-induced surface membrane damage in male adult *Schistosoma mansoni* BH strain worms. [*Exp Parasitol.*](https://www.ncbi.nlm.nih.gov/pubmed/24768955/) 2014 Jul;142:83-90. doi: 10.1016/j.exppara.2014.04.010.; Bertão, H.G., da Silva, R.A.R., Padilha, R.J.R. et al. Ultrastructural analysis of miltefosine-induced surface membrane damage in adult *Schistosoma mansoni* BH strain worms. *Parasitol Res* (2012) 110: 2465. https://doi.org/10.1007/s00436-011-2786-5.

4- *The order and description of the results session is very confusing to the reader;*

R- We expect the Referee to agree that we have improved the order and description of the results session in the revised text.

5- *In the Results session: "Phenotypic effects of NJ series compounds of S. mansoni schistosomula" (page 17). The description is very poor and confused;*

R- Again, we expect the Referee to agree that we have improved the order and description of the results session in the revised text "Phenotypic effects of NJ series compounds of *S. mansoni* schistosomula" (page 13).

6- *In the Results session:”Fig 2. Synergistic effect of NJ05 + NJ07 on schistosomula viability (A and B) ATP” (page 17). It is extremely premature to describe this result as a synergistic effect of NJ05 + NJ07 compounds, considering that pharmacokinetic and pharmacodynamics mechanisms does not yet been studied. Please review this affirmation with caution;*

R- According to the Referee’s request we have now replaced the term “synergistic effect of NJ05 + NJ07” by “combinatorial effect of NJ05 + NJ07” in the text (page 13).

7*- Finally, in the results session "Assessment by quantitative PCR of the expression of genes involved with vitellaria and female reproduction" (page 23).*

*Whereas: "Tested genes involved in egg biosynthesis were all confirmed by qPCR as downregulated in females treated with 25 μM NJ05 for 2 days (Fig 8), with a significant reduction in expression of p14, Tyrosinase 2, p48 and fs800 ".*

*Suggestion: It would be very interesting to observe the eggs viability by miracid hatching assay. e.g. Sarvel, A.K. Mem Inst Oswaldo Cruz (2006), 101 (Suppl. I): 289-292.*

R- We agree that this is a very interesting idea given by the Referee. Unfortunately, we cannot perform these experiments at this moment. We will be able to conduct these experiments in future work.

**Reviewer #2:** REVIEWER COMMENTS ON THE MANUSCRIPT PONE – D – 19- 21297

*The research article entitled “In vitro activity of aryl-thiazole derivatives against Schistosoma mansoni schistosomula and adult worms” reports the synthesis and the antischistosomal characterization of a series of aryl-thiazoles.*

*The article has distinction when regarding the quantity and quality of the biological assays performed as well as about the synthetic approach and the choice of this chemical class. It lacks, however, the important selectivity information of the compounds since, nowadays, to prove effectivity of a compound are not enough to establish the potential of it to be employed as a lead or even as a hit compound in drug discovery.*

*No mention about any kind of selectivity assays is present in this study. At least a cytotoxicity assay against some usual cell lines should be conducted, such as VERO, HaCAT or lymphocyte cells.*

R- As requested by the Referee, we have now added on page 17 the results of cytotoxicity evaluation of NJ05, NJ07 and NJ05 + NJ07 on HEK293 cell line (Human Embryo Kidney- HEK-293 -ATCC® CRL-1573™) and HES cell line (endometrial epithelial cell line, kindly provided by Prof. Douglas Anthony Kniss - Ohio State University, USA). Also, in the Discussion (pages 24 and 25) we added a last paragraph where we comment on the differential cytotoxicity between the two cell lines.

*I also suggest, to a further submission, a careful review of the manuscript in the following aspects:*

*MATERIAL AND METHODS*

*Page 6: Even the synthetic approach being well-known, it would be kind if the authors provided some important aspects of the synthesis, such as the molar proportion between the reagents and the molar excess of sodium acetate employed. Everyone who reads your article could, easily, reproduce the synthesis directly from it. Yet, regarding the reaction media, did the authors employ any solvent that allowed the reflux condition? If yes, please, provide this information along the experimental text.*

*I also seem as important to include some characterization information about the compounds, such as NMR and IR signals, melting point ranges, etc. The journal public include chemists which would like to be provided with this information. No spectra needed, however. Only the attributions would be enough.*

R- The molar quantities that were used for the reagents in each reaction were now added to the revised text (pages 6 and 7). In addition, physicochemical and spectroscopic data were now entered for all compounds (pages 7-9).

*RESULTS*

*Page 11: Figure 2. Remember to mention that the data did not correspond only to NJ05+NJ07 together but also to NJ05 and NJ07 individually. The Figure 2 title did not reflect the exhibited data.*

R- We would like to apologize for that mistake, and we have now changed the title of the figure with the appropriate information (page 13).

*Page 13: When discussing about decreasing in worm motility, it seems that NJ05+NJ07 together leaded to a higher decrease than NJ05 and NJ07 alone. Similarly to the discussed by the authors about the viability being decreased only by the NJ05, it seemed, to me, that the association decreases in motility was similar to the observed with NJ07 only. Please, review this data.*

R- We thank the Referee for bringing up this subject. Indeed, as you stated, the motility results for NJ05 and NJ05+NJ07 were similar and this was not clear in the text. We have now changed the text as follows: “*S. mansoni* adult worms exhibited a larger decrease in motility when exposed to NJ05 and NJ05 + NJ07, compared with NJ07 treatment alone. This phenotype was dependent upon concentration and incubation time (Fig 3D – 3F). After three days with the lowest concentration tested (12.5 µM), NJ05 and NJ05 + NJ07 decreased the motility to 40 – 50 % compared with controls (Fig 3D and 3F), while NJ07 treatment at the same concentration led to a reduction of only 15-20 % in motility (Fig 3E).” (pages 15-16)

*Page 14: Authors present the results of NJ07 on the worm pairing and oviposition. But, at the end of such discussion, they mention that the other compounds were able to result in impairments of 75 to 90%. So, these compounds were better in modulate the worm pairing than NJ07 and that is exactly what Figure D from S1 text informs. For instance, NJ03 is better than NJ07 at 50 μM. So, why the authors have chosen NJ07 to discuss along the text and presented the better data in supplementary material?
It that is not the case, please review the phrase construction which induces the reader to understand what I stated.*

R- We have chosen NJ05 and NJ07 as the best compounds for describing the other phenotypes because both of them are the best ones when considering all the observed parameters together (i.e., viability, pairing, oviposition and motility in adult worms and also viability for schistosomula). As you have correctly stated, looking only at pairing the results with NJ03 were better than with other compounds; however, the results were only better for this parameter (pairing), and also at the highest concentration tested, which is not comparable to the results found in NJ05 and NJ07 treatment. When considering the other assessed phenotypes, the NJ03 compound was not the best one.

*DISCUSSION*

*Page 19: Authors constructed a parallel between their findings and the best ways to achieve antischistosomal activity:*

*“In light of the parasite biology and according to the evaluation of these parameters, schistosomicidal activity can be effective essentially in three different ways: causing schistosomula death, thus preventing host infection, inhibiting oviposition that could lead to a decrease in the pathological phenotype of schistosomiasis (hepato- and splenomegaly), and causing adult worm’s death [44, 45]. All three of the above schistosomicidal effects were present in our in vitro assays with NJ compounds…”*

*Exactly by the fact that several different actions were observed, SELECTIVITY ASSAYS must be conducted, since it could be possible the existence of different mechanisms of actions which, in turn, could lead to potential side effects. This should be better explored by the authors and is a fundamental step to be accomplished to this article to be accepted.*

R- We agree with the Referee that different mechanisms of action could lead to potential side effects. Therefore, as a first assessment of the potential side effects of the compounds here tested, we have now added on page 17 the results of cytotoxicity evaluation of NJ05, NJ07 and NJ05 + NJ07 on HEK293 cell line (Human Embryo Kidney- HEK-293 -ATCC® CRL-1573™) and HES cell line (endometrial epithelial cell line, kindly provided by Prof. Douglas Anthony Kniss - Ohio State University, USA). Also, in the Discussion (pages 24 and 25) we added a last paragraph where we comment on the differential cytotoxicity between the two cell lines.

*Page 19: Authors did the following affirmation:*

*“Trimethoxylated derivatives (NJ04, NJ05, NJ07 and NJ11) showed a better profile in reducing the viability of S. mansoni schistosomula and adult worms. The main electronic change on the molecule caused by tri-substitution is an increase of the electronic density, and this effect significantly increased the schistosomicidal activity, also demonstrated by the synergic effect of compounds NJ05 + NJ07 which differ only by 4-nitro and 4-bromo substituents, respectively.”*

*It is dangerous to correlates the action to the increase of the electronic density reached with the trimethoxy series, especially when this was the only aspect discussed. Electronic effects could not be the only ones involved in the compound’s behavior. The 3-OMe group, for instance, could be an important docking point to these compounds in a potential receptor. The right group at the right position could made an enormous difference in compounds activity. This is particularly true for hydrogen bond interaction, which could, for instance, be involved in the 3-MeO group (present only in the trimethoxy series) recognition by the worm’s target. Please, review this statement.*

*It is true that the trimethoxy aromatic group has already being proved to be important for different antiparasitic activities, but not only by its electronic contributions.*

R- We would like to thank the Referee for the above considerations and indeed it is not the best way to describe the correlation between the phenotypes observed and electronic density. Thus, we have now revised this paragraph as follows:

“One important chemical property of methoxy substituent is that its oxygen atom is an acceptor of proton for different types of hydrogen-bonds [46] favoring the biological activity of new compounds by improving the target recognition [47]. Besides, trimethoxy-substitution increases the electronic density, and this could also be related to the higher schistosomicidal activity of this class compared with the dimethoxylated ones. The results corroborate with studies involving other parasites such as Plasmodium falciparum [48], Leishmania braziliensis [49] and Trypanosoma cruzi [50], evidencing the importance of methoxy group trisubstituted aromatic nuclei for antiparasitic activity.” (page 22, middle of first paragraph)

*Pages 19-20: Authors composed the hypothesis that “NJ compounds might have caused oxidative stress and the consequent biological activity observed here.” Then they correlate the compound’s action to a possible direct amino acid nitrosylation of SmTGR.*

*The problem is that nitrosylation processes are related to nitroso group (NO) and not to nitro group itself (NO2). Nitro group usually suffers reduction up to the hydroxylamine and, further, to amine producing reactive oxygen and nitrogen species which, in turn, destroy DNA and other important cell subunits. It is an unspecific mechanism of action which is also related, sometimes, to unsuitable toxicity profiles. Nitrosylation, on the other hand, implies the presence of a NO species or a NO donor species which, nitroaromatic systems such as the one studied here, do not produce in vivo.*

*Please, review this hypothesis.*

R- We agree with the Referee that the hypothesis was wrong, and that nitrosylation should not have been mentioned in our context. We have therefore removed the sentence and the reference. (see page 22, second paragraph)

*Pages 20: In the statement “Permeation in biological membranes may have been fundamental for the good activity presented by NJ07.” Be careful!*

*4-Cl would have been more active if membrane permeation would be such significant aspect since its hydrophobic contribution ( of Hansch = 0.70) is higher than that obtained with the 4-NO2 group ( of Hansch = 0.24) present in NJ05 structure. You have limited experimental data to comment on the relationship between chemical structure and biological activity by now. Some comments would be interesting but always being careful to highlight the fact that further studies to explore other structural aspects should be performed.*

R- Following the Referee’s comments, we have now removed the statement questioned by him, namely *“Permeation in biological membranes may have been fundamental for the good activity presented by NJ07.”* In its place, the following sentences were added:

“The effects of para-substituents on biological activity can be explained by the hydrophobicity (π) and electronic effects (σ) as stated by Craig, 1971 [58]. Both NO2 and Bromo substituents present positive electronic (+σ) and lipophilic (+π) effects. This means inferior availability of free electron pairs, which could promote membrane permeation.” (page 23, first paragraph)

Also, at the end of the paragraph the following sentence was added: “Further studies are necessary to explore other structural aspects of these compounds besides their possible lipophilicity contribution to membrane permeabilization.” (page 23, first paragraph)

*Minor revisions include a careful review of the phrases constructions which are a little bit strange to the formal English.*

R- Thank you for your observation and we have revised the phrases constructions in the manuscript.

**Reviewer #3:**

*In this manuscript the authors report their work on synthesizing a series of thiazole derivatives and the assessment of the anthelmintic activity of 8 of these compounds in vitro. I feel that this description of their work is very thorough and their study design draws upon all of the best molecular techniques available to synthesize, interrogate and execute these particular class of studies. Their assessments of parasite motility and mortality, egg laying, adult worm pairing and parasite viability clearly demonstrate the antischistosomal activity of their compounds, especially compounds NJ05 and NJ07. The antischistosomal activity of compound NJ05 against juvenile worms shows particular promise given the lack of activity by the gold standard of treatment, praziquantel. The only reason for my suggestion of minor revision include some grammatical issues in the text (e.g. verb tense).*

 R- Thank you for your comments and we have revised the grammatical issues in the manuscript.
